# Information Theoretic Security for Broadcasting of Two Encrypted Sources under Side-Channel Attacks †

**DOI:** 10.3390/e21080781

**Published:** 2019-08-09

**Authors:** Bagus Santoso, Yasutada Oohama

**Affiliations:** Department of Computer and Network Engineering, University of Electro-Communications, Tokyo 182-8585, Japan

**Keywords:** information theoretic security, side-channel attacks, Shannon cipher system, one helper source coding problem, strong converse theorem

## Abstract

In this paper, we propose a theoretical framework to analyze the secure communication problem for broadcasting two encrypted sources in the presence of an adversary which launches side-channel attacks. The adversary is not only allowed to eavesdrop the ciphertexts in the public communication channel, but is also allowed to gather additional information on the secret keys via the side-channels, physical phenomenon leaked by the encryption devices during the encryption process, such as the fluctuations of power consumption, heat, or electromagnetic radiation generated by the encryption devices. Based on our framework, we propose a countermeasure against such adversary by using the post-encryption-compression (PEC) paradigm, in the case of one-time-pad encryption. We implement the PEC paradigm using affine encoders constructed from linear encoders and derive the explicit the sufficient conditions to attain the exponential decay of the information leakage as the block lengths of encrypted sources become large. One interesting feature of the proposed countermeasure is that its performance is independent from the type of side information leaked by the encryption devices.

## 1. Introduction

In recent years, it has become very common that one person holds multiple wireless communication devices and broadcasts the messages through multiple devices. In order to ensure secrecy, it is a standard practice to encrypt the data before broadcasting them into the public communication channel. The usual security problem that is considered in such system of broadcasting encrypted sources is the secrecy against an adversary which eavesdrops the ciphertexts sent via the public communication channel. However, Kocher et al. [1,2] have shown that an adversary may also learn “side” information about the secret keys from “side-channel”, i.e., the measurements of physical phenomenon that occur in the physical devices where the encryption procedures are implemented. Such adversary is called as side-channel adversary. Examples of the physical phenomenon exploited by the side-channel adversaries are the fluctuations of time cost [1], the fluctuations of power consumption [2], and the electromagnetic (EM) radiation [3]. In this paper, we are focusing on a specific scenario where an adversary is not only eavesdropping on the public communication channel but is also launching side-channel attacks on multiple communication devices owned by a sender. We consider that this kind of side-channel attack is feasible in the real world when multiple devices owned by the sender relocated in the same area such that the adversary can catch the side information from the devices directly.

### 1.1. Modelling Side-Channel Attacks

**Figure 1:**
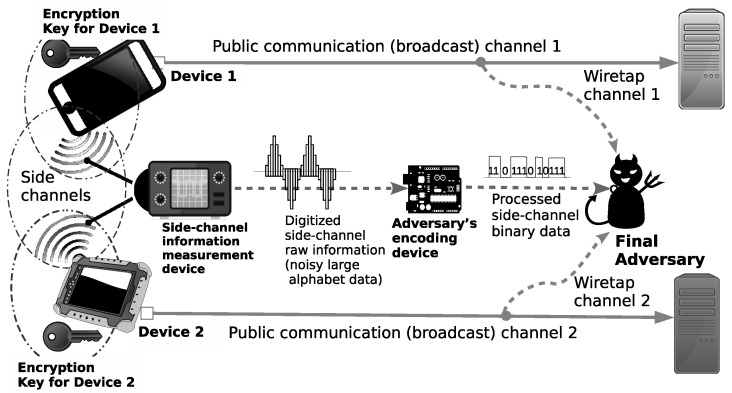
Side-channel attacks in a broadcasting system.

The adversarial/security model we use in this paper and its relation to a real-world example are shown in Figure 1. Basically, we adapt the approach in [4] on modeling the side-channel, where the side-channel is modelled as a rate constraint noiseless channel.

We describe our model in a more formal way as follows. Let us consider two sources $X_{1}$ and $X_{2}$, where each is encrypted in two different encryption devices using secret keys $K_{1}$ and $K_{2}$, respectively, resulting in ciphertexts $C_{1}$ and $C_{2}$, respectively. The ciphertexts $C_{1}$ and $C_{2}$ are sent by the sender to multiple receivers through multiple public communication channels. The adversary A is allowed to obtain: (1) ciphertexts $C_{1}$ and $C_{2}$ from the public communication channels, and also (2) “noisy” digital data *Z* generated by the probe or the measurement device from the physical phenomenon leaked by all encryption devices of the sender. The measurement device may just be a simple analog-to-digital converter that converts the analog data representing physical information leaked by the devices into “noisy” digital data *Z*. In our model, we represent the measurement process as a communication channel *W*.The adversary A is equipped with a side-channel encoding device $\varphi_{A}$ which encodes and processes *Z* into the binary data $M_{A}$. Finally, combining $C_{1}$, $C_{2}$, and $M_{A}$, A will attempt to derive information on the sources $X_{1}$ and $X_{2}$.

### 1.2. Our Results and Methodology in Brief

We show that we can strengthen the secrecy/security of the Shannon ciphers which are implemented on multiple physical devices of a sender in a broadcasting system against an adversary who collects ciphertexts and launches side-channel attacks by a simple method of reencoding the ciphertexts before releasing them into the public communication channels. This method is based on post-encryption-compression (PEC) paradigm. We prove that, in the case that all encryption devices implement one time pad encryption, we can strengthen the secrecy/security using appropriate affine encoders $\varphi_{1}$ and $\varphi_{2}$ which transform the original ciphertexts $C_{1}$ and $C_{2}$ into reencoded ciphertexts ${\tilde{C}}_{1}$ and ${\tilde{C}}_{2}$.

More formally, we prove that, for any distribution of the secret keys ($K_{1}$, $K_{2}$) and any measurement device (used to convert the physical information from a side-channel into the noisy large alphabet data *Z*), we can derive an achievable rate region for ($R_{1}$, $R_{2}$, $R_{A}$), where $R_{1}$ and $R_{2}$ are the encoding rates of $\varphi_{1}$ and $\varphi_{2}$, respectively, $R_{A}$ is the encoding rate of adversary’s encoding device $\varphi_{A}$. More precisely, if we reencode $C_{1}$ and $C_{2}$ into ${\tilde{C}}_{1}$ and ${\tilde{C}}_{2}$ using $\varphi_{1}$ and $\varphi_{2}$ with encoding rates $R_{1}$ and $R_{2}$, respectively, such that $R_{1}$ and $R_{2}$ are inside the achievable region, then we can attain reliability and security in the following sense:

- anyone with secret keys $K_{1}$ and $K_{2}$ can construct appropriate decoders that decrypt and encode the reencoded ciphertexts ${\tilde{C}}_{1}$ and ${\tilde{C}}_{2}$ into original sources $X_{1}$ and $X_{2}$ with exponentially decaying error probability, and
- the amount of information on the sources $X_{1}$ and $X_{2}$ gained by any adversary A which collects the reencoded ciphertexts $C_{1}$, $C_{2}$ the encoded side-channel information $M_{A}$ is exponentially decaying to zero as long as the side-channel encoding device $\varphi_{A}$ encodes *Z* into $M_{A}$ with the rate $R_{A}$ which is inside the achievable rate region.

Taking the advantage of the homomorphic property of one-time-pad and affine encoding, we separate the theoretical analysis of reliability and security such that we can deal with each issue independently. For reliability analysis, similar to the analysis in [4,5,6,7], we mainly obtain our result by adapting the result of Csizár [8] on the universal coding using linear codes. Our main theorem on security is based on the technique developed in [4] which is actually a combination of two other techniques. One is a technique developed by Oohama in [9] for deriving approximation error exponents for the intrinsic randomness problem in some framework of distributed random number extraction. (This technique is is also used in the security analysis in Santoso and Oohama [6,10].) Another one is a technique proposed by Oohama [11] to establish exponential strong converse theorem for the one helper source coding problem. (This technique is used in the security analysis for the side channel attacks to the Shannon cipher system.)

In addition, since we model the side-channel as a rate constraint noiseless channel, all theoretical results in this paper are independent from the type of side-channel information the adversary collects from the encryption devices. This means that the countermeasure we propose in this paper can be applied against any type of side-channel attacks launched by the adversary, e.g., timing attacks, electromagnetic radiation or power analysis, and so on.

### 1.3. Related Works

The use of PEC for communication system can be traced back to the work by Johnson et al., in [12]. However, their main focus is the issue of reliability and they only provide weak secrecy for security, whereas, in this paper, we provide security based on the strong secrecy [13,14].

Several theoretical models analyzing the security of a cryptographic system against side-channel attacks have been proposed in the literature. However, most of the existing works are applicable only for specific characteristics of the leaked physical information. For example, Brier et al. [15] and Coron et al. [16] propose a statistical model for side-channel attacks using the information from power consumption and the running time, whereas Agrawal et al. [3] propose a statistical model for side-channel attacks using electromagnetic (EM) radiations. A more general model for side-channel attacks is proposed by Köpf et al. [17] and Backes et al. [18], but they are heavily dependent upon implementation on certain specific devices. Micali et al. [19] propose a very general security model to capture the side-channel attacks, but they fail to offer any hint of how to build a concrete countermeasure against the side-channel attacks. One of the closest existing models to ours is the general framework for analyzing side-channel attacks proposed by Standaert et al. [20]. However, the authors of [20] propose a countermeasure against side-channel attacks that is different from ours, i.e., noise insertion on implementation. It should be noted that the noise insertion countermeasure proposed by [20] depends on the characteristics of the leaked physical information. Another model that is similar to ours in the sense that it is independent from the type of leaked physical information is proposed by Chérisey et al. [21,22]. However, the main aim of [21,22] is only establishing the mathematical link between success probability of side-channel adversary and mutual information and no countermeasure is proposed.

### 1.4. Organization of This Paper

This paper is structured as follows. In Section 2, we show the basic notations and definitions that we use throughout this paper, and we also describe the formal formulations of our model and the security problem. In Section 3, we explain the idea and the formulation of our proposed solution. In Section 4, we state our main theorem on the reliability and security of our solution. In Section 5, we show the proof of our main theorem. In Section 6, we discuss an alternative formulation of our model and problem. In Section 7, we show the comparison between our current results in this paper and our previous works. We put our conclusions in Section 9. We put the proofs of other related propositions, lemmas, and theorems in the appendix.

## 2. Problem Formulation

### 2.1. Preliminaries

In this subsection, we show the basic notations and related consensus used in this paper.

*Random Source of Information and Key:* For each i=1,2, let $X_{i}$ be a random variable from a finite set $X_{i}$. For each i=1,2, let ${\left\{X_{i,t}\right\}}_{t=1}^{\infty }$ be two stationary discrete memoryless sources (DMS) such that, for each t=1,2,…, $X_{i,t}$ take values in finite set $X_{i}$ and has the same distribution as that of $X_{i}$ denoted by $p_{X_{i}}={\left\{p_{X_{i}}\left(x_{i}\right)\right\}}_{x_{i}\in X_{i}}$. The stationary DMS ${\left\{X_{i,t}\right\}}_{t=1}^{\infty },$ are specified with $p_{X_{i}}$.

We next define the two keys used in the two common cryptosystems. For each i=1,2, let $\left(K_{1},K_{2}\right)$ be a pair of two correlated random variables taken from the same finite set $X_{1}\times X_{2}$. Let ${\left\{\left(K_{1,t},K_{2,t}\right)\right\}}_{t=1}^{\infty }$ be a stationary discrete memoryless source such that, for each t=1,2,…, $\left(K_{1,t},K_{2,t}\right)$ takes values in $X_{1}\times$ $X_{2}$ and has the same distribution as that of $\left(K_{1},K_{2}\right)$ denoted by

$$p_{K_{1}K_{2}}={\left\{p_{K_{1}K_{2}}\left(k_{1},k_{2}\right)\right\}}_{\left(k_{1},k_{2}\right)\in X_{1}\times X_{2}}.$$

The stationary DMS $\{{\left(K_{1,t},K_{2,t}\right\}}_{t=1}^{\infty }$ is specified with $p_{K_{1}K_{2}}$.

*Random Variables and Sequences:* We write the sequence of random variables with length *n* from the information sources as follows: $X_{i}^{n}:=X_{i,1}X_{i,2}\cdots X_{i,n},i=1,2$. Similarly, the strings with length *n* of $X_{i}^{n}$ are written as $x_{i}^{n}:=x_{i,1}x_{i,2}\cdots x_{i,n}\in X_{i}^{n}$. For $\left(x_{1}^{n},x_{2}^{n}\right)\in X_{1}^{n}\times X_{2}^{n}$, $p_{X_{1}^{n}X_{2}^{n}}\left(x_{1}^{n},x_{2}^{n}\right)$ stands for the probability of the occurrence of $\left(x_{1}^{n},x_{2}^{n}\right)$. When the information source is memoryless specified with $p_{X_{1}X_{2}}$, we have the following equation holds:

$$p_{X_{1}^{n}X_{2}^{n}}\left(x_{1}^{n},x_{2}^{n}\right)=\prod_{t=1}^{n}p_{X_{1}X_{2}}\left(x_{1,t},x_{2,t}\right).$$

In this case, we write $p_{X_{1}^{n}X_{2}^{n}}\left(x_{1}^{n},x_{2}^{n}\right)$ as $p_{X_{1}X_{2}}^{n}\left(x_{1}^{n},x_{2}^{n}\right)$. Similar notations are used for other random variables and sequences.

*Consensus and Notations:* Without loss of generality, throughout this paper, we assume that $X_{1}$ and $X_{2}$ are finite fields. The notation ⊕ is used to denote the field addition operation, while the notation ⊖ is used to denote the field subtraction operation, i.e., a⊖b=a⊕(−b) for any elements a,b from the same finite field. All discussions and theorems in this paper still hold although $X_{1}$ and $X_{2}$ are different finite fields. However, for the sake of simplicity, we use the same notation for field addition and subtraction for both $X_{1}$ and $X_{2}$. Throughout this paper, all logarithms are taken to the natural basis.

### 2.2. Basic System Description

In this subsection, we explain the basic system setting and basic adversarial model we consider in this paper. First, let the information source and the key be generated independently by three different parties $S_{\mathrm{gen},1}$, $S_{\mathrm{gen},2}$ and $K_{\mathrm{gen}}$, respectively. In our setting, we assume the following:

- The random keys $K_{1}^{n}$ and $K_{2}^{n}$ are generated by $K_{\mathrm{gen}}$ from uniform distribution. We may have a correlation between $K_{1}^{n}$ and $K_{2}^{n}$.
- The sources $X_{1}^{n}$ and $X_{2}^{n}$, respectively, are generated by $S_{\mathrm{gen},1}$ and $S_{\mathrm{gen},2}$. Those are independent from the keys.

**Figure 2:**
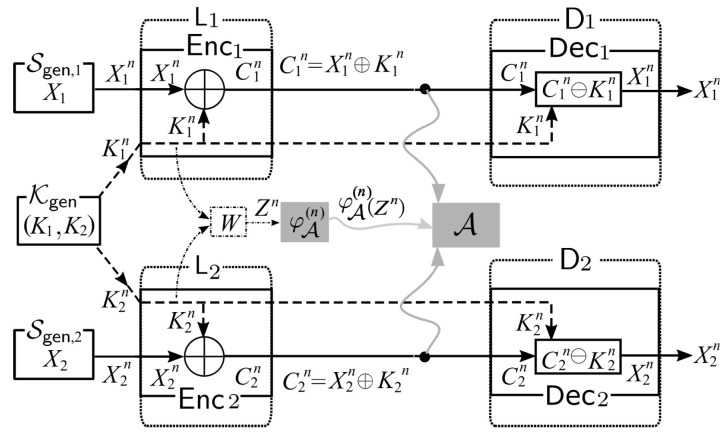
Side-channel attacks to the two Shannon cipher systems.

Next, let the two random sources $X_{1}^{n}$ and $X_{2}^{n}$, respectively, from $S_{\mathrm{gen},1}$ and $S_{\mathrm{gen},2}$ be sent to two separated nodes $L_{1}$ and $L_{2}$. In addition, let two random key (sources) $K_{1}^{n}$ and $K_{2}^{n}$ from $K_{\mathrm{gen}}$ be also sent separately to $L_{1}$ and $L_{2}$. Further settings of our system are described as follows. Those are also shown in Figure 2.

1. *Separate Sources Processing:* For each i=1,2, at the node $L_{i}$, $X_{i}^{n}$ is encrypted with the key $K_{i}^{n}$ using the encryption function ${\mathrm{Enc}}_{i}$. The ciphertext $C_{i}^{n}$ of $X_{i}^{n}$ is given by $C_{i}^{n}:={\mathrm{Enc}}_{i}\left(X_{i}^{n}\right)=X_{i}^{n}⊕K_{i}^{n}.$
2. *Transmission:* The ciphertexts $C_{1}^{n}$ and $C_{2}^{n}$, respectively, are sent to the information processing center $D_{1}$ and $D_{2}$ through two *public* communication channels. Meanwhile, the keys $K_{1}^{n}$ and $K_{2}^{n}$, respectively are sent to $D_{1}$ and $D_{2}$ through two *private* communication channels.
3. *Sink Nodes Processing:* For each i=1,2, in $D_{i}$, we decrypt the ciphertext $C_{i}^{n}$ using the key $K_{i}^{n}$ through the corresponding decryption procedure ${\mathrm{Dec}}_{i}$ defined by ${\mathrm{Dec}}_{i}\left(C_{i}^{n}\right)=C_{i}^{n}⊖K_{i}^{n}$. It is obvious that we can correctly reproduce the source output $X^{n}$ from $C_{i}^{n}$ and $K_{i}^{n}$ by the decryption function ${\mathrm{Dec}}_{i}$.

*Side-Channel Attacks by Eavesdropper Adversary*: An *(eavesdropper) adversary*A eavesdrops on the public communication channel in the system. The adversary A also uses a side information obtained by side-channel attacks. Let Z be a finite set and let $W:X_{1}\times X_{2}\to Z$ be a noisy channel. Let *Z* be a channel output from *W* for the input random variable *K*. We consider the discrete memoryless channel specified with *W*. Let $Z^{n}\in Z^{n}$ be a random variable obtained as the channel output by connecting $\left(K_{1}^{n},K_{2}^{n}\right)\in X_{1}^{n}\times X_{2}^{n}$ to the input of channel. We write a conditional distribution on $Z^{n}$ given $\left(K_{1}^{n},K_{2}^{n}\right)$ as

$$W^{n}={\left\{W^{n}\left(z^{n}|k_{1}^{n},k_{2}^{n}\right)\right\}}_{\left(k_{1}^{n},k_{2}^{n},z^{n}\right)\in X_{1}^{n}\times X_{2}^{n}\times Z^{n}}.$$

Since the channel is memoryless, we have

(1) $$W^{n}\left(z^{n}|k_{1}^{n},k_{2}^{n}\right)=\prod_{t=1}^{n}W\left(z_{t}|k_{1,t},k_{2,t}\right).$$

On the above output $Z^{n}$ of $W^{n}$ for the input $\left(K_{1}^{n},K_{2}^{n}\right)$, we assume the following:

- The two random pairs $\left(X_{1},X_{2}\right)$, $\left(K_{1},K_{2}\right)$ and the random variable *Z*, satisfy $\left(X_{1},X_{2}\right)⊥\left(K_{1},K_{2},Z\right)$, which implies that $\left(X_{1}^{n},X_{2}^{n}\right)⊥\left(K_{1}^{n},K_{2}^{n},Z^{n}\right)$.
- By side-channel attacks, the adversary A can access $Z^{n}$.

We next formulate side information the adversary A obtains by side-channel attacks. For each n=1,2,⋯, let $\varphi_{A}^{\left(n\right)}:Z^{n}\to M_{A}^{\left(n\right)}$ be an encoder function. Set $\varphi_{A}:={\left\{\varphi_{A}^{\left(n\right)}\right\}}_{n=1,2,\cdots }.$ Let

$$R_{A}^{\left(n\right)}:=\frac{1}{n}\log||\varphi_{A}\left|\right|=\frac{1}{n}\log\left|M_{A}^{\left(n\right)}\right|$$

be a rate of the encoder function $\varphi_{A}^{\left(n\right)}$. For $R_{A}>0$, we set

$$F_{A}^{\left(n\right)}\left(R_{A}\right):=\left\{\varphi_{A}^{\left(n\right)}:R_{A}^{\left(n\right)}\leq R_{A}\right\}.$$

On encoded side information, the adversary A obtains, we assume, the following:

- The adversary A, having accessed $Z^{n}$, obtains the encoded additional information $\varphi_{A}^{\left(n\right)}\left(Z^{n}\right)$. For each n=1,2,⋯, the adversary A can design $\varphi_{A}^{\left(n\right)}$.
- The sequence ${\left\{R_{A}^{\left(n\right)}\right\}}_{n=1}^{\infty }$ must be upper bounded by a prescribed value. In other words, the adversary A must use $\varphi_{A}^{\left(n\right)}$ such that, for some $R_{A}$ and for any sufficiently large *n*, $\varphi_{A}^{\left(n\right)}\in F_{A}^{\left(n\right)}\left(R_{A}\right)$.

**Figure 3:**
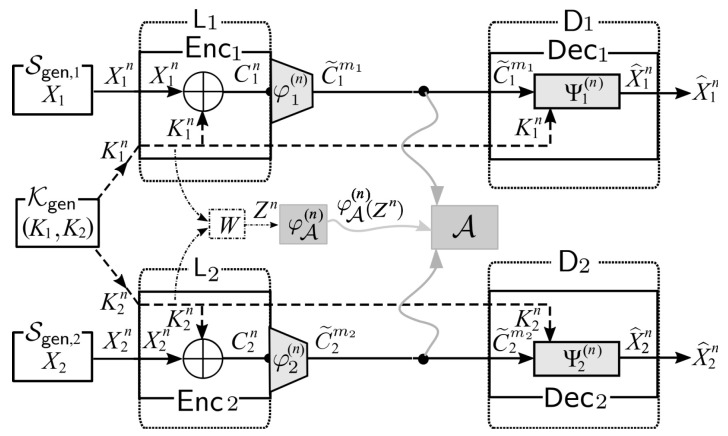
Sys: a system of broadcast encryption with post-encryption coding.

As a solution to the side channel attacks, we consider a system of broadcast encryption with post-encryption coding. We call this system as Sys. The illustration of Sys is shown in Figure 3.

1. *Encoding at Source node $L_{i},i=1,2$:* For each i=1,2, we first use $\varphi_{i}^{\left(n\right)}$ to encode the ciphertext $C_{i}^{n}=X_{i}^{n}⊕K_{i}^{n}$. A formal definition of $\varphi_{i}^{\left(n\right)}$ is $\varphi_{i}^{\left(n\right)}:$ $X_{i}^{n}\to$ $X_{i}^{m_{i}}$. Let ${\tilde{C}}_{i}^{m_{i}}=\varphi_{i}^{\left(n\right)}\left(C_{i}^{n}\right)$. Instead of sending $C_{i}^{n}$, we send ${\tilde{C}}_{i}^{m_{i}}$ to the public communication channel.
2. *Decoding at Sink Nodes $D_{i},i=1,2$:* For each i=1,2, $D_{i}$ receives ${\tilde{C}}_{i}^{m_{i}}$ from a public communication channel. Using common key $K_{i}^{n}$ and the decoder function $\Psi_{i}^{\left(n\right)}:X_{i}^{m}\times X_{i}^{n}\to X_{i}^{n}$, $D_{i}$ outputs an estimation ${\hat{X}}_{i}^{n}=\Psi_{i}^{\left(n\right)}\left({\tilde{C}}_{i}^{m_{i}},K_{i}^{n}\right)$ of $X_{i}^{n}$.

*On Reliability and Security*: From the description of our system in the previous section, the decoding process in our system above is successful if ${\hat{X}}_{i}^{n}=X_{i}^{n}$ holds. Combining this and Equation (5), it is clear that the decoding error probabilities $p_{e,i},i=1,2,$ are as follows:

$$\begin{array}{ll} p_{e,i}= & p_{e}\left(\varphi_{i}^{\left(n\right)},\Psi_{i}^{\left(n\right)}|p_{X_{i}}^{n}\right):=\mathrm{Pr}\left[\Psi_{i}^{\left(n\right)}\left(\varphi_{i}^{\left(n\right)}\left(X_{i}^{n}\right)\right)\neq X_{i}^{n}\right]. \end{array}$$

Set $M_{A}^{\left(n\right)}=\varphi_{A}^{\left(n\right)}\left(Z^{n}\right)$. The information leakage $\Delta^{\left(n\right)}$ on $\left(X_{1}^{n},X_{2}^{n}\right)$ from $\left({\tilde{C}}_{1}^{m_{1}},{\tilde{C}}_{2}^{m_{2}},M_{A}^{\left(n\right)}\right)$ is measured by the mutual information between $\left(X_{1}^{n},X_{2}^{n}\right)$ and $({\tilde{C}}_{1}^{m_{1}},{\tilde{C}}_{2}^{m_{2}},$ $M_{A}^{\left(n\right)})$. This quantity is formally defined by

$$\begin{array}{l} \Delta^{\left(n\right)}=\Delta^{\left(n\right)}\left(\varphi_{1}^{\left(n\right)},\varphi_{2}^{\left(n\right)},\varphi_{A}^{\left(n\right)}|p_{X_{1}X_{2}}^{n},p_{ZK_{1}K_{2}}^{n}\right):=I\left(X_{1}^{n}X_{2}^{n};{\tilde{C}}_{1}^{m_{2}},{\tilde{C}}_{2}^{m_{2}},M_{A}^{\left(n\right)}\right). \end{array}$$

*Reliable and Secure Framework*:

**Definition** **1.** *A pair $\left(R_{1},R_{2}\right)$ is achievable under $R_{A}$>0 for the system Sys if there exists two sequences $\{(\varphi_{i}^{\left(n\right)},$$\Psi_{i}^{\left(n\right)}{)\}}_{n\geq 1},i=1,2,$ such that ∀ϵ>0, $\exists n_{0}=n_{0}\left(\epsilon \right)\in N_{0}$, $\forall n\geq n_{0}$, we have for i=1,2,*

$$\begin{array}{l} \frac{1}{n}\log|X_{i}^{m_{i}}|=\frac{m_{i}}{n}\log\left|X_{i}\right|\leq R_{i},p_{e}\left(\varphi_{i}^{\left(n\right)},\Psi_{i}^{\left(n\right)}|p_{X_{i}}^{n}\right)\leq \epsilon , \end{array}$$

*and for any eavesdropper A with $\varphi_{A}$ satisfying $\varphi_{A}^{\left(n\right)}\in F_{A}^{\left(n\right)}\left(R_{A}\right)$, we have*

$$\begin{array}{l} \Delta^{\left(n\right)}\left(\varphi_{1}^{\left(n\right)},\varphi_{2}^{\left(n\right)},\varphi_{A}^{\left(n\right)}|p_{X_{1}X_{2}}^{n},p_{ZK_{1}K_{2}}^{n}\right)\leq \epsilon . \end{array}$$

**Definition** **2** **(Reliable** **and** **Secure** **Rate** **Region).** *Let $R_{\mathrm{Sys}}(p_{X_{1}X_{2}},$$p_{ZK_{1}K_{2}})$ denote the set of all $\left(R_{A},R\right)$ such that R is achievable under $R_{A}$. We call $R_{\mathrm{Sys}}\left(p_{X_{1}X_{2}},p_{ZK_{1}K_{2}}\right)$ the***reliable and secure rate***region*.

**Definition** **3.** *A five tuple $\left(R_{1},R_{2},E_{1},E_{2},F\right)$ is achievable under $R_{A}>0$ for the system Sys if there exists a sequence $\{(\varphi_{i}^{\left(n\right)},$$\Psi_{i}^{\left(n\right)}{)\}}_{n\geq 1}$, i=1,2, such that ∀ϵ>0, $\exists n_{0}=n_{0}\left(\epsilon \right)\in N_{0}$, ∀n$\geq n_{0}$, we have for i=1,2,*

$$\begin{array}{l} \frac{1}{n}\log|X_{i}^{m_{i}}|=\frac{m_{i}}{n}\log\left|X_{i}\right|\leq R_{i},p_{e}\left(\varphi_{i}^{\left(n\right)},\Psi_{i}^{\left(n\right)}|p_{X_{i}}^{n}\right)\leq e^{-n(E_{i}-\epsilon )}, \end{array}$$

*and for any eavesdropper A with $\varphi_{A}$ satisfying $\varphi_{A}^{\left(n\right)}\in F_{A}^{\left(n\right)}\left(R_{A}\right)$, we have*

$$\begin{array}{l} \Delta^{\left(n\right)}\left(\varphi_{1}^{\left(n\right)},\varphi_{2}^{\left(n\right)},\varphi_{A}^{\left(n\right)}|p_{X_{1}X_{2}}^{n},p_{ZK_{1}K_{2}}^{n}\right)\leq e^{-n(F-\epsilon )}. \end{array}$$

**Definition** **4** **(Rate,** **Reliability,** **and** **Security** **Region).** *Let $D_{\mathrm{Sys}}(p_{X_{1}X_{2}},$$p_{K_{1}K_{2}},W)$ denote the set of all $\left(R_{A},R,E,F\right)$ such that $\left(R_{1},R_{2},E_{1},E_{2},F\right)$ is achievable under $R_{A}$. We call $D_{\mathrm{Sys}}(p_{X_{1}X_{2}},$$p_{K_{1}K_{2}},W)$ the***rate, reliability, and security***region*.

## 3. Proposed Idea: Affine Encoder as Privacy Amplifier

For each n=1,2,⋯, let $\phi_{i}^{\left(n\right)}:X_{i}^{n}\to X_{i}^{m_{i}}$ be a linear mapping. We define the mapping $\phi_{i}^{\left(n\right)}$ by

(2) $$\phi_{i}^{\left(n\right)}\left(x_{i}^{n}\right)=x_{i}^{n}A_{i}\;\mathrm{for}\;x_{i}^{n}\in X_{i}^{n},$$

where $A_{i}$ is a matrix with *n* rows and $m_{i}$ columns. Entries of $A_{i}$ are from $X_{i}$. We fix $b_{i}^{m_{i}}\in X_{i}^{m_{i}}$. Define the mapping $\varphi_{i}^{\left(n\right)}:X_{i}^{n}\to X_{i}^{m_{i}}$ by

$$\begin{array}{ll} \varphi_{i}^{\left(n\right)}\left(k_{i}^{n}\right):= & \phi_{i}^{\left(n\right)}\left(k_{i}^{n}\right)⊕b_{i}^{m_{i}}=k_{i}^{n}A_{i}⊕b_{i}^{m_{i}},\;\mathrm{for}\;k_{i}^{n}\in X_{i}^{n}. \end{array}$$

The mapping $\varphi_{i}^{\left(n\right)}$ is called the affine mapping induced by the linear mapping $\phi_{i}^{\left(n\right)}$ and constant vector $b_{i}^{m_{i}}$ $\in X_{i}^{m_{i}}$. By the definition of $\varphi_{i}^{\left(n\right)}$, the following affine structure holds:

(3) $$\begin{array}{cc} \varphi_{i}^{\left(n\right)}\left(x_{i}^{n}⊕k_{i}^{n}\right) & =\left(x_{i}^{n}⊕k_{i}^{n}\right)A_{i}⊕b_{i}^{m_{i}}=x_{i}^{n}A_{i}⊕\left(k_{i}^{n}A_{i}⊕b_{i}^{m_{i}}\right)\\ & =\phi_{i}^{\left(n\right)}\left(x_{i}^{n}\right)⊕\varphi_{i}^{\left(n\right)}\left(k_{i}^{n}\right),\;\mathrm{for}\;x_{i}^{n},k_{i}^{n}\in X_{i}^{n}. \end{array}$$

Next, let $\psi_{i}^{\left(n\right)}$ be the corresponding decoder for $\phi_{i}^{\left(n\right)}$ such that $\psi_{i}^{\left(n\right)}:X_{i}^{m_{i}}\to X_{i}^{n}.$ Note that $\psi_{i}^{\left(n\right)}$ does not have a linear structure in general.

*Description of Proposed Procedure*: We describe the procedure of our privacy amplified system as follows:

1. *Encoding at Source node $L_{i},i=1,2$:* First, we use $\varphi_{i}^{\left(n\right)}$ to encode the ciphertext $C_{i}^{n}=X_{i}^{n}⊕K_{i}^{n}$ Let ${\tilde{C}}_{i}^{m_{i}}=\varphi_{i}^{\left(n\right)}\left(C_{i}^{n}\right)$. Then, instead of sending $C^{n}$, we send ${\tilde{C}}_{i}^{m_{i}}$ to the public communication channel. By the affine structure (3) of encoder, we have that
  (4) $$\begin{array}{ll} {\tilde{C}}_{i}^{m_{i}}=\varphi_{i}^{\left(n\right)}\left(X_{i}^{n}⊕K_{i}^{n}\right) & =\phi_{i}^{\left(n\right)}\left(X_{i}^{n}\right)⊕\varphi_{i}^{\left(n\right)}\left(K_{i}^{n}\right)={\tilde{X}}_{i}^{m_{i}}⊕{\tilde{K}}_{i}^{m_{i}}, \end{array}$$
  where we set ${\tilde{X}}_{i}^{m_{i}}:=\phi_{i}^{\left(n\right)}\left(X_{i}^{n}\right),{\tilde{K}}_{i}^{m_{i}}:=\varphi_{i}^{\left(n\right)}\left(K_{i}^{n}\right).$
2. *Decoding at Sink Node $D_{i},i=1,2$:* First, using the linear encoder $\varphi_{i}^{\left(n\right)}$, $D_{i}$ encodes the key $K_{i}^{n}$ received through private channel into ${\tilde{K}}_{i}^{m_{i}}=$ $\varphi_{i}^{\left(n\right)}\left(K_{i}^{n}\right)$. Receiving ${\tilde{C}}_{i}^{m_{i}}$ from public communication channel, $D_{i}$ computes ${\tilde{X}}_{i}^{m_{i}}$ in the following way. From (4), we have that the decoder $D_{i}$ can obtain ${\tilde{X}}_{i}^{m_{i}}$ $=\phi_{i}^{\left(n\right)}\left(X_{i}^{n}\right)$ by subtracting ${\tilde{K}}_{i}^{m_{i}}=\varphi_{i}^{\left(n\right)}\left(K_{i}^{n}\right)$ from ${\tilde{C}}_{i}^{m_{i}}$. Finally, $D_{i}$ outputs ${\hat{X}}_{i}^{n}$ by applying the decoder $\psi_{i}^{\left(n\right)}$ to ${\tilde{X}}_{i}^{m_{i}}$ as follows:
  (5) $$\begin{array}{ll} {\hat{X}}_{i}^{n} & =\psi_{i}^{\left(n\right)}\left({\tilde{X}}_{i}^{m_{i}}\right)=\psi_{i}^{\left(n\right)}\left(\phi_{i}^{\left(n\right)}\left(X_{i}^{n}\right)\right). \end{array}$$

**Figure 4:**
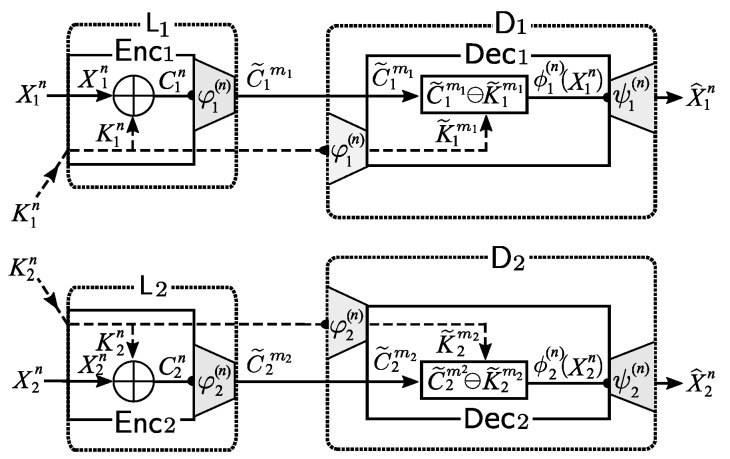
Our proposed countermeasure: affine encoders as privacy amplifiers.

Our privacy amplified system described above is illustrated in Figure 4.

## 4. Main Results

In this section, we state our main results. To describe our results, we define several functions and sets. Let *U* be an auxiliary random variable taking values in a finite set U. We assume that the joint distribution of $\left(U,Z,K_{1},K_{2}\right)$ is

$$p_{UZK_{1}K_{2}}\left(u,z,k_{1},k_{2}\right)=p_{U}\left(u\right)p_{Z|U}\left(z|u\right)p_{K_{1}K_{2}|Z}\left(k_{1},k_{2}|z\right).$$

The above condition is equivalent to $U↔Z↔(K_{1},K_{2})$. In the following argument for convenience of descriptions of definitions, we use the following notations:

$$\begin{array}{l} R_{3}:=R_{1}+R_{2},X_{3}:=X_{1}\times X_{2},k_{3}:=\left(k_{1},k_{2}\right),K_{3}:=\left(K_{1},K_{2}\right). \end{array}$$

For each i=1,2,3, we simply write $p_{i}=p_{UZK_{i}}$. Specifically, for i=3, we have $p_{3}=p_{UZK_{1}K_{2}}=p$. Define the three sets of probability distribution with i=1,2,3:

(6) $$\begin{array}{ll} P(p_{ZK_{i}}):= & \{p_{UZK_{i}}:|U|\leq |Z|+1,U↔Z↔K_{i}\}. \end{array}$$

For i=1,2,3, let us define as follows:

(7) $$\begin{array}{ll} R_{i}\left(p_{i}\right) & :=\begin{array}{c} \{\left(R_{A},R_{i}\right):R_{A},R_{i}\geq 0,\;\;R_{A}\geq I_{}\left(Z;U\right),R_{i}\geq H_{}\left(K_{i}|U\right)\}, \end{array} \end{array}$$

(8) $$\begin{array}{cc} R_{i}\left(p_{ZK_{i}}\right) & :=\underset{p_{i}\in P\left(p_{ZK_{i}}\right)}{⋃}R_{i}\left(p_{i}\right). \end{array}$$

The two regions $R_{i}\left(p_{ZK_{i}}\right),i=1,2$ have the same form as the region appearing as the admissible rate region in the one-helper source coding problem posed and investigated by Ahlswede and Körner [23]. We can show that the region $R_{i}\left(p_{ZK_{i}}\right),i=1,2,$ and $R_{3}\left(p_{ZK_{1}K_{2}}\right)$ satisfy the following property.

**Property** **1.**

*(a)*  *The region $R_{i}\left(p_{ZK_{i}}\right),i=1,2$ is a closed convex subset of $R_{+}^{2}$. The region $R_{3}\left(p_{ZK_{1}K_{2}}\right)$ is a closed convex subset of $R_{+}^{3}$*.
*(b)*  *The bound |U|≤|Z|+1 is sufficient to describe $R_{i}\left(p_{ZK_{i}}\right),i=1,2,3$*.

We define several quantities to state our main result. Let i∈{1,2}. We first define a function related to an exponential upper bound of $p_{e}\left(\phi_{i}^{\left(n\right)},\psi_{i}^{\left(n\right)}|p_{X_{i}}^{n}\right)$. Let ${\bar{X}}_{i}$ be an arbitrary random variable over $X_{i}$ and has a probability distribution $p_{{\bar{X}}_{i}}$. Let $P(X_{i})$ denote the set of all probability distributions on $X_{i}$. For $R_{i}\geq 0$ and $p_{X_{i}}\in$ $P(X_{i})$, we define the following function:

(9) $$\begin{array}{ll} E(R_{i}|p_{X_{i}}) & :=\min_{p_{{\bar{X}}_{i}}\in P\left(X_{i}\right)}\{{\left[R_{i}-H\left({\bar{X}}_{i}\right)\right]}^{+}+D(p_{{\bar{X}}_{i}}\left|\right|p_{X_{i}})\}. \end{array}$$

We next define a function related to an exponential upper bound of $\Delta^{\left(n\right)}\left(\varphi_{1}^{\left(n\right)},\varphi_{2}^{\left(n\right)},\varphi_{A}^{\left(n\right)}|p_{X_{1}X_{2}}^{n},p_{ZK_{1}K_{2}}^{n}\right)$. For each i=1,2,3, we define three sets of probability distributions on U ×Z $\times X_{i}$ by

$$\begin{array}{ll} \tilde{P}\left(p_{ZK_{i}}\right):= & \{p=p_{UZK_{i}}:|U|\leq |Z|,U↔Z↔K_{i}\}. \end{array}$$

Furthermore, for each i=1,2,3, we define three sets of probability distributions on U ×Z $\times X_{i}$ by

$$\begin{array}{l} Q\left(p_{K_{i}|Z}\right):=\left\{q_{i}=q_{UZK_{i}}:q_{K_{i}Z|U}=p_{K_{i}Z|U}:\mathrm{for}\;\mathrm{some}p_{i}\in \tilde{P}\left(p_{ZK_{i}}\right)\right\}. \end{array}$$

For each i=1,2,3, for $\left(\mu ,\alpha \right)\in {\left[0,1\right]}^{2}$, and for $q_{i}=q_{UZK_{i}}\in Q\left(p_{K_{i}|Z}\right)$, define

ωqi|pZ(μ,α)(z,ki|u):=α¯logqZ(z)pZ(z)+αμlogqZ|U(z|u)pZ(z)+μ¯log1qKi|U(ki|u),Ω(μ,α)(qi|pZ):=−logEqexp−ωqi|pZ(μ,α)(Z,Ki|U),Ω(μ,α)(pZKi):=minqi∈Q(pKi|Z)Ω(μ,α)(qi|pZ),F(μ,α)(μRA+μ¯Ri|pZKi):=Ωi(μ,α)(pKi,W)−α(μRA+μ¯Ri)2+αμ¯,F(RA,Ri|pZKi):=sup(μ,α)∈[0,1]2F(μ,α)(μRA+μ¯Ri|pZKi).

In [11] (extended version), Oohama proved several properties on $F(R_{A},R_{i}|p_{ZK_{i}}),i=1,2,3$. According to [11] (extended version), we have the following property.

**Property** **2.** *For any i=1,2,3 and for any $\tau \in (0,\left(1/2\right)\rho \left(p_{ZK_{i}}\right))$, the condition $(R_{A},$$R_{i}+\tau )\notin R_{i}\left(p_{ZK_{i}}\right)$ implies*

$$\begin{array}{l} F\left(R_{A},R_{i}|p_{ZK_{i}}\right)>\frac{\rho (p_{ZK_{i}})}{4}\cdot g^{2}\left(\frac{\tau }{\rho (p_{ZK_{i}})}\right)>0, \end{array}$$

*where $\rho (p_{ZK_{i}}),i=1,2,3$, respectively, are some quantities depending on $p_{ZK_{i}}$ and g is the inverse function of $\vartheta \left(a\right):=a+\left(5/4\right)a^{2},a\;\geq 0$*.

Let us define as follows:

(10) $$\begin{array}{l} F_{\min}\left(R_{A},R_{1},R_{2}|p_{ZK_{1}K_{2}}\right):=\min_{i=1,2,3}F\left(R_{A},R_{i}|p_{ZK_{i}}\right). \end{array}$$

Our main result is as follows.

**Theorem** **1.** *For any $R_{A},R_{1},R_{2}>0$ and any $p_{ZK_{1}K_{2}}$, there exists two sequence of mappings ${\left\{\left(\varphi_{i}^{\left(n\right)},\psi_{i}^{\left(n\right)}\right)\right\}}_{n=1}^{\infty },i=1,2$ such that, for any $p_{X_{i}},i=1,2,$ and any $n\geq {\left(R_{1}+R_{2}\right)}^{-1}$, we have*

(11) $$\begin{array}{ll} \frac{1}{n}\log\left|X_{i}^{m_{i}}\right| & =\frac{m_{i}}{n}\log\left|X_{i}\right|\leq R_{i},\\ p_{e}\left(\phi_{i}^{\left(n\right)},\psi_{i}^{\left(n\right)}|p_{X_{i}}^{n}\right) & \leq e^{-n[E\left(R_{i}|p_{X_{i}}\right)-\delta_{i,n}]},i=1,2 \end{array}$$

*and for any eavesdropper A with $\varphi_{A}$ satisfying $\varphi_{A}^{\left(n\right)}\in F_{A}^{\left(n\right)}\left(R_{A}\right)$, we have*

(12) $$\begin{array}{ll} \Delta^{\left(n\right)}\left(\varphi_{1}^{\left(n\right)},\varphi_{2}^{\left(n\right)},\varphi_{A}^{\left(n\right)}|p_{X_{1}X_{2}}^{n},p_{K_{1}K_{2}}^{n},W^{n}\right) & \leq e^{-n[F_{\min}\left(R_{A},R_{1},R_{2}|p_{ZK_{1}K_{2}}\right)-\delta_{3,n}]}, \end{array}$$

*where $\delta_{i,n},i=1,2,3$ are defined by*

$$\begin{array}{ll} \delta_{i,n}:= & \frac{1}{n}\log\left[e{\left(n+1\right)}^{2|X_{i}|}\right.\times \left.\left\{1+{\left(n+1\right)}^{|X_{1}|}+{\left(n+1\right)}^{|X_{2}|}\right\}\right],for\;i=1,2,\\ \delta_{3,n}:= & \frac{1}{n}\log\left[15n\left(R_{1}+R_{2}\right)\times \left\{1+{\left(n+1\right)}^{|X_{1}|}+{\left(n+1\right)}^{|X_{2}|}\right\}\right]. \end{array}$$

*Note that, for i=1,2,3, $\delta_{i,n}\to 0$ as n→∞*.

Detail of the proof of Theorem 1 will be explained in Section 5.

The functions $E(R_{i}|p_{X_{i}})$ and $F(R_{A},R_{1},R_{2}|p_{ZK_{1}K_{2}})$ take positive values if $\left(R_{A},R_{1},R_{2}\right)$ belongs to the set

$$\begin{array}{ll} R_{\mathrm{Sys}}^{\left(\mathrm{in}\right)}\left(p_{X_{1}X_{2}},p_{ZK_{1}K_{2}}\right) & :=\left\{R_{1}>H\left(X_{1}\right)\right\}\cap \left\{R_{2}>H\left(X_{2}\right)\right\}\underset{i=1,2,3}{⋂}R_{i}^{c}\left(p_{ZK_{i}}\right). \end{array}$$

Thus, by Theorem 1, under $\left(R_{A},R_{1},R_{2}\right)\in R_{\mathrm{Sys}}^{\left(\mathrm{in}\right)}(p_{X_{1}X_{2}},$ $p_{ZK_{1}K_{2}}),$ we have the following:

- On the reliability, for i=1,2, $p_{e}\left(\phi_{i}^{\left(n\right)},\psi_{i}^{\left(n\right)}|p_{X_{i}}^{n}\right)$ goes to zero exponentially as *n* tends to infinity, and its exponent is lower bounded by the function $E(R_{i}|p_{X_{i}})$.
- On the security, for any $\varphi_{A}$ satisfying $\varphi_{A}^{\left(n\right)}\in$ $F_{A}^{\left(n\right)}\left(R_{A}\right)$, the information leakage $\Delta^{\left(n\right)}(\varphi_{1}^{\left(n\right)},\varphi_{2}^{\left(n\right)},\varphi_{A}^{\left(n\right)}$ $|p_{X_{1}X_{2}}^{n},p_{ZK_{1}K_{2}}^{n})$ on $X_{1}^{n},X_{2}^{n}$ goes to zero exponentially as *n* tends to infinity, and its exponent is lower bounded by the function $F_{\min}\left(R_{A},R_{1},R_{2}|p_{ZK_{1}K_{2}}\right)$.
- For each i=1,2, any code $\left(\phi_{i}^{\left(n\right)},\psi_{i}^{\left(n\right)}\right)$ that attains the exponent function E( $R_{i}\left|p_{X_{i}}\right)$ is a universal code that depends only on $R_{i}$ not on the value of the distribution $p_{X_{i}}$.

Define

$$\begin{array}{ll} D_{\mathrm{Sys}}^{\left(\mathrm{in}\right)}\left(p_{X_{1}X_{1}},p_{ZK_{1}K_{2}}\right) & :=\{\left(R_{A},R_{1},R_{2},E\left(R_{1}|p_{X_{1}}\right),E\left(R_{2}|p_{X_{2}}\right),F_{\min}\left(R_{A},R_{1},R_{2}|p_{K_{1}K_{2}}\right)\right):\\ & \;\left(R_{1},R_{2}\right)\in R_{\mathrm{Sys}}^{\left(\mathrm{in}\right)}\left(p_{X_{1}X_{2}},p_{ZK_{1}K_{2}}\right)\}. \end{array}$$

From Theorem 1, we obtain the following corollary:

**Corollary** **1.** $$\begin{array}{l} R_{\mathrm{Sys}}^{\left(\mathrm{in}\right)}\left(p_{X_{1}X_{1}},p_{ZK_{1}K_{2}}\right)\subseteq R_{\mathrm{Sys}}\left(p_{X_{1}X_{1}},p_{ZK_{1}K_{2}}\right),\\ D_{\mathrm{Sys}}^{\left(\mathrm{in}\right)}\left(p_{X_{1}X_{1}},p_{ZK_{1}K_{2}}\right)\subseteq D_{\mathrm{Sys}}\left(p_{X_{1}X_{1}},p_{ZK_{1}K_{2}}\right). \end{array}$$

**Remark** **1.** *Note that, from the definitions of sets $P(p_{ZK_{i}})$, $R_{i}\left(p_{ZK_{i}}\right)$, it is easy to see that the set $R_{\mathrm{Sys}}^{\left(\mathrm{in}\right)}\left(p_{X_{1}X_{1}},p_{ZK_{1}K_{2}}\right)$ is the intersection of the outer regions of all possible adversarial encoding of A (where each encoding is represented by one auxiliary variable U) within rate $R_{A}$. Moreover, since we use the strong converse theorem developed in [11] instead of the weak converse, we can guarantee that in $R_{\mathrm{Sys}}^{\left(\mathrm{in}\right)}\left(p_{X_{1}X_{1}},p_{ZK_{1}K_{2}}\right)$, not only the adversarial decoding success probability, but also the information leakage decays to zero at an exponential rate*.

**Remark** **2.** *Thanks to the separation between reliability and security analysis, the results related security in this paper will still hold even in the case where the sources are correlated. Moreover, our proposed countermeasure can strengthen the secrecy even in the case where the marginal distribution of each key $K_{i}$, i.e., $p_{K_{i}}$, (i=1,2) is not uniform*.

### Examples of Extremal Cases

In the remaining part of this section, we give two simple examples of $R_{\mathrm{Sys}}^{\left(\mathrm{in}\right)}\left(p_{X_{1}X_{1}},p_{ZK_{1}K_{2}}\right)$. Those correspond to extremal cases on the correlation of $\left(K_{1},K_{2},Z\right)$. In those two examples, we assume that $X_{1}=X_{2}=\left\{0,1\right\}$ and $p_{X_{1}}\left(1\right)=s_{1},p_{X_{2}}\left(1\right)=s_{2}$. We further assume that $p_{K_{1},K_{2}}$ has the binary symmetric distribution given by

$$p_{K_{1}K_{2}}\left(k_{1},k_{2}\right)=\left(1/2\right)\left[\bar{\rho }k_{1}⊕k_{2}+\rho \bar{k_{1}⊕k_{2}}\right]\mathrm{for}\left(k_{1},k_{2}\right)\in {\left\{0,1\right\}}^{2},$$

where ρ∈[0,0.5] is a parameter indicating the correlation level of $\left(K_{1},K_{2}\right)$.

**Example** **1.** *We consider the case where $W=p_{Z|K_{1}K_{2}}$ is given by*

$$\begin{array}{cc} W(z|k_{1},k_{2})= & W\left(z|k_{1}\right)=\bar{\rho_{A}}k_{1}⊕z+\rho_{A}\bar{k_{1}⊕z}\;for\left(k_{1},k_{2},z\right)\in {\left\{0,1\right\}}^{3}. \end{array}$$

*In this case, we have $K_{2}↔K_{1}↔Z$. This corresponds to the case where the adversary A attacks only node $L_{1}$. Let $N_{A}$ be a binary random variable with $p_{N_{A}}\left(1\right)=\rho_{A}$. We assume that $N_{A}$ is independent from $\left(X_{1},X_{2}\right)$ and $\left(K_{1},K_{2}\right)$. Using $N_{A}$, Z can be written as $Z=K_{1}⊕N_{A}.$ The inner bound for this example denoted by $R_{\mathrm{Sys},\mathrm{ex}1}^{\left(\mathrm{in}\right)}\left(p_{X_{1}X_{2}},p_{ZK_{1}K_{2}}\right)$ is the following:*

(13) $$\begin{array}{ll} R_{\mathrm{Sys},\mathrm{ex}1}^{\left(\mathrm{in}\right)}\left(p_{X_{1}X_{2}},p_{ZK_{1}K_{2}}\right)=\{\left(R_{A},R_{1},R_{2}\right): & 0\leq R_{A}\leq \log2-h\left(\theta \right),\\ h\left(s_{1}\right)<R_{1} & <h(\rho_{A}*\theta ),\\ h\left(s_{2}\right)<R_{2} & <h\left((\rho *\rho_{A})*\theta \right),\\ R_{1}+R_{2} & <h\left(\rho \right)+h\left(\rho_{A}*\theta \right)for\;some\;\theta \in \left[0,1\right]\}, \end{array}$$

*where h(·) denotes the binary entropy function and $a*b:=a\bar{b}+\bar{a}b$*.

One can easily compute $R_{\mathrm{Sys},\mathrm{ex}1}^{\left(\mathrm{in}\right)}\left(p_{X_{1}X_{2}},p_{ZK_{1}K_{2}}\right)$ based on the solution for the problem of lossless source coding with helper, which is explained in [24]. The computation of $R_{\mathrm{Sys},\mathrm{ex}1}^{\left(\mathrm{in}\right)}\left(p_{X_{1}X_{2}},p_{ZK_{1}K_{2}}\right)$ is given in Appendix A.

**Example** **2.** *We consider the case of ρ=0.5. In this case, $K_{1}$ and $K_{2}$ is independent. In this case, we have no information leakage if $R_{A}=0$. We assume that $W=p_{Z|K_{1}K_{2}}$ is given by*

$$\begin{array}{cc} W(z|k_{1},k_{2})= & \bar{\rho_{A}}k_{1}⊕k_{2}⊕z+\rho_{A}\bar{k_{1}⊕k_{2}⊕z}\;for\left(k_{1},k_{2},z\right)\in {\left\{0,1\right\}}^{3}. \end{array}$$

*Let $N_{A}$ be the same random variable as the previous example. Using $N_{A}$, Z can be written as $Z=K_{1}⊕K_{2}⊕N_{A}.$ The inner bound in this example denoted by $R_{\mathrm{Sys},\mathrm{ex}2}^{\left(\mathrm{in}\right)}($$p_{X_{1}X_{2}},p_{ZK_{1}K_{2}})$ is the following:*

(14) $$\begin{array}{ll} R_{\mathrm{Sys},\mathrm{ex}2}^{\left(\mathrm{in}\right)}\left(p_{X_{1}X_{2}},p_{ZK_{1}K_{2}}\right)=\{\left(R_{A},R_{1},R_{2}\right):0 & \leq R_{A}\leq \log2-h\left(\theta \right),\\ h\left(s_{i}\right)<R_{i} & <\log2,i=1,2,\\ R_{1}+R_{2} & <\log2+h\left(\rho_{A}*\theta \right)for\;some\;\theta \in \left[0,1\right]\}. \end{array}$$

Similar to Example 1, one can also easily compute $R_{\mathrm{Sys},\mathrm{ex}2}^{\left(\mathrm{in}\right)}\left(p_{X_{1}X_{2}},p_{ZK_{1}K_{2}}\right)$ based on the solution for the problem of lossless source coding with helper, which is explained in [24]. Computation of $R_{\mathrm{Sys},\mathrm{ex}2}^{\left(\mathrm{in}\right)}\left(p_{X_{1}X_{2}},p_{ZK_{1}K_{2}}\right)$ is given in Appendix B.

**Figure 5:**
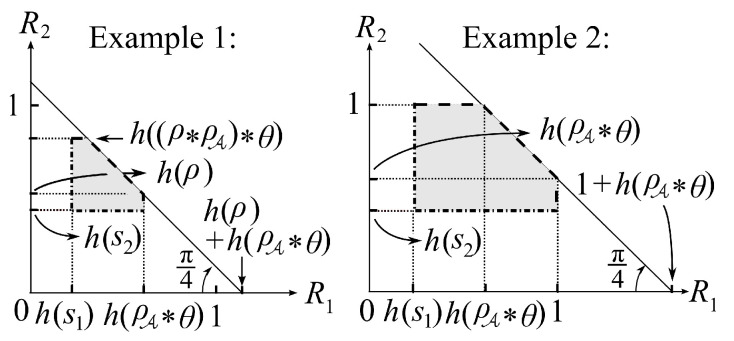
Shape of the regions $R_{\mathrm{Sys},\mathrm{ex}i}^{\left(\mathrm{in}\right)}($ $p_{X_{1}X_{2}},p_{ZK_{1}K_{2}})$ $\cap \{R_{A}=1-h\left(\theta \right)\}$,i=,1,2.

For the above two examples, we show the section of the regions $R_{\mathrm{Sys},\mathrm{exi}}^{\left(\mathrm{in}\right)}($ $p_{X_{1}X_{2}},p_{ZK_{1}K_{2}})$ for i=1,2 by the plane $\{R_{A}=\log2-h\left(\theta \right)\},$ which is shown in Figure 5.

## 5. Proofs of the Main Results

In this section, we prove Theorem 1.

### 5.1. Types of Sequences and Their Properties

In this subsection, we prepare basic results on the types. Those results are basic tools for our analysis of several bounds related to error provability of decoding or security.

**Definition** **5.** *For each i=1,2 and for any n-sequence $x_{i}^{n}=x_{i,1}x_{i,2}\cdots$$x_{i,n}\in X^{n}$, $n(x_{i}|x_{i}^{n})$ denotes the number of t such that $x_{i,t}=x_{i}$. The relative frequency ${\left\{n(x_{i}|x_{i}^{n})/n\right\}}_{x_{i}\in X_{i}}$ of the components of $x_{i}^{n}$ is called the type of $x_{1}^{n}$ denoted by $P_{x^{n}}$. The set that consists of all the types on X is denoted by $P_{n}\left(X\right)$. Let ${\bar{X}}_{i}$ denote an arbitrary random variable whose distribution $P_{{\bar{X}}_{i}}$ belongs to $P_{n}\left(X_{i}\right)$. For $p_{{\bar{X}}_{i}}\in P_{n}\left(X_{i}\right)$, set*

$$T_{{\bar{X}}_{i}}^{n}:=\left\{x_{i}^{n}:\;P_{x_{i}^{n}}=p_{{\bar{X}}_{i}}\right\}.$$

For set of types and joint types, the following lemma holds. For the detail of the proof, see Csiszár and Körner [25].

**Lemma** **1.**

*(a)*  $\begin{array}{c} |P_{n}\left(X_{i}\right)|\leq {\left(n+1\right)}^{|X_{i}|}. \end{array}$
*(b)*  *For $P_{{\bar{X}}_{i}}\in P_{n}\left(X_{i}\right)$*,
  $$\begin{array}{ll} {\left(n+1\right)}^{-|X_{i}|}e^{nH({\bar{X}}_{i})} & \leq |T_{{\bar{X}}_{i}}^{n}|\leq e^{nH({\bar{X}}_{i})}. \end{array}$$
*(c)*  *For $x_{i}^{n}\in T_{{\bar{X}}_{i}}^{n}$*,
  $$\begin{array}{ll} p_{X_{i}}^{n}\left(x_{i}^{n}\right) & =e^{-n[H\left({\bar{X}}_{i}\right)+D(p_{{\bar{X}}_{i}}\left|\right|p_{X_{i}})]}. \end{array}$$

By Lemma 1 parts (b) and (c), we immediately obtain the following lemma:

**Lemma** **2.** *For $p_{{\bar{X}}_{i}}\in P_{n}\left(X_{i}\right)$*,

$$\begin{array}{l} p_{X_{i}}^{n}\left(T_{{\bar{X}}_{i}}^{n}\right)\leq e^{-nD(p_{{\bar{X}}_{i}}||p_{X_{i}})}. \end{array}$$

### 5.2. Upper Bounds on Reliability and Security

In this subsection, we evaluate upper bounds of $p_{e}(\phi_{i}^{\left(n\right)},$ $\psi_{i}^{\left(n\right)}\left|p_{X_{i}}^{n}\right),$ i=1,2, and $\Delta_{n}\left(\varphi_{1}^{\left(n\right)},\varphi_{2}^{\left(n\right)},\varphi_{A}^{\left(n\right)}\right|p_{X_{1}X_{2}}^{n},p_{ZK_{1}}$ ${}_{K_{2}Un})$. For $p_{e}(\phi_{i}^{\left(n\right)}$, $\psi_{i}^{\left(n\right)}\left|p_{X_{i}}^{n}\right)$, we derive an upper bound that can be characterized with a quantity depending on $\left(\phi_{i}^{\left(n\right)},\psi_{i}^{\left(n\right)}\right)$ and type $P_{x_{i}^{n}}$ of sequences $x_{i}^{n}\in X_{i}^{n}$. We first evaluate $p_{e}\left(\phi_{i}^{\left(n\right)},\psi_{i}^{\left(n\right)}|p_{X_{i}}^{n}\right),i=1,2$. For $x_{i}^{n}\in X_{i}^{n}$ and $p_{\bar{X}}\in P_{n}\left(X_{i}\right),$ we define the following functions:

$$\begin{array}{ll} \Xi_{x_{i}^{n}}\left(\phi_{i}^{\left(n\right)},\psi_{i}^{\left(n\right)}\right) & :=\left\{\begin{array}{ccc} 1 & \; & \mathrm{if}\;\psi_{i}^{\left(n\right)}\left(\phi_{i}^{\left(n\right)}\left(x_{i}^{n}\right)\right)\neq x_{i}^{n},\;\\ 0 & \; & \mathrm{otherwise}, \end{array}\right.\\ \Xi_{{\bar{X}}_{i}}\left(\phi^{\left(n\right)},\psi^{\left(n\right)}\right) & :=\frac{1}{|T_{{\bar{X}}_{i}}^{n}|}\sum_{x_{i}^{n}\in T_{{\bar{X}}_{i}}^{n}}\Xi_{x_{i}^{n}}\left(\phi_{i}^{\left(n\right)},\psi_{i}^{\left(n\right)}\right). \end{array}$$

Then, we have the following lemma.

**Lemma** **3.** *In the proposed system, for i=1,2 and for any pair of $(\phi_{i}^{\left(n\right)},$$\psi_{i}^{\left(n\right)})$, we have*

(15) $$\begin{array}{ll} p_{e}\left(\phi_{i}^{\left(n\right)},\psi_{i}^{\left(n\right)}|p_{X_{i}}^{n}\right) & \leq \sum_{p_{{\bar{X}}_{i}}\in P_{n}\left(X_{i}\right)}\Xi_{\bar{X}}\left(\phi_{i}^{\left(n\right)},\psi_{i}^{\left(n\right)}\right)e^{-nD(p_{{\bar{X}}_{i}}||p_{X_{i}})}. \end{array}$$

Proof of this lemma is found in [26]. We omit the proof.

We next discuss upper bounds of

$$\begin{array}{l} \Delta_{n}\left(\varphi_{1}^{\left(n\right)},\varphi_{2}^{\left(n\right)},\varphi_{A}^{\left(n\right)}|p_{X_{1}X_{2}}^{n},p_{ZK_{1}K_{2}}^{n}\right)=I\left({\tilde{C}}_{1}^{m_{1}}{\tilde{C}}_{2}^{m_{2}},M_{A}^{\left(n\right)};X_{1}^{n}X_{2}^{n}\right). \end{array}$$

On an upper bound of $I({\tilde{C}}_{1}^{m_{1}}{\tilde{C}}_{2}^{m_{2}},M_{A}^{\left(n\right)};X_{1}^{n}X_{2}^{n})$, we have the following lemma:

**Lemma** **4.** (16) $$\begin{array}{ll} I({\tilde{C}}_{1}^{m_{1}}{\tilde{C}}_{2}^{m_{2}},M_{A}^{\left(n\right)};X_{1}^{n}X_{2}^{n}) & \leq \left.\left.\left.\;\;\;D\left(p_{K_{1}^{m_{1}}K_{2}^{m_{2}}|M_{A}^{\left(n\right)}}\right|\right|p_{V_{1}^{m_{1}}V_{2}^{m_{2}}}\right|p_{M_{A}^{\left(n\right)}}\right), \end{array}$$

*where $p_{V_{1}^{m_{1}}V_{2}^{m_{2}}}$ represents the uniform distribution over $X_{1}^{m_{1}}\times$$X_{2}^{m_{2}}$*.

We can prove Lemma 4 using a similar method shown in [4]. The detailed proof is given in Appendix C.

### 5.3. Random Coding Arguments

We construct a pair of affine encoders $\left(\varphi_{1}^{\left(n\right)},\varphi_{2}^{\left(n\right)}\right)$ using the random coding method. For the two decoders $\psi_{i}^{\left(n\right)},i=1,2$, we propose the minimum entropy decoder used in Csiszár [8] and Oohama and Han [27].

*Random Construction of Affine Encoders*: For each i=1,2, we first choose $m_{i}$ such that

$$m_{i}:=\left\lfloor \frac{nR_{i}}{\log|X_{i}|}\right\rfloor ,$$

where ⌊a⌋ stands for the integer part of *a*. It is obvious that, for i=1,2,

$$R_{i}-\frac{1}{n}\leq \frac{m_{i}}{n}\log\left|X_{i}\right|\leq R_{i}.$$

By the Definition (2) of $\phi_{i}^{\left(n\right)}$, we have that, for $x_{i}^{n}\in X_{i}^{n}$,

$$\begin{array}{l} \phi_{i}^{\left(n\right)}\left(x_{i}^{n}\right)=x_{i}^{n}A_{i}, \end{array}$$

where $A_{i}$ is a matrix with *n* rows and $m_{i}$ columns. By the definition (2) of $\varphi_{i}^{\left(n\right)}$, we have that, for $k_{i}^{n}\in X_{i}^{n}$,

$$\begin{array}{l} \varphi_{i}^{\left(n\right)}\left(k_{i}^{n}\right)=k_{i}^{n}A_{i}+b_{i}^{m_{i}}, \end{array}$$

where for each i=1,2, $b_{i}^{m_{i}}$ is a vector with $m_{i}$ columns. Entries of $A_{i}$ and $b_{i}^{m_{i}}$ are from the field of $X_{i}$. Those entries are selected at random, independently from each other and with uniform distribution. Randomly constructed linear encoder $\phi_{i}^{\left(n\right)}$ and affine encoder $\varphi_{i}^{\left(n\right)}$ have three properties shown in the following lemma.

**Lemma** **5** **(Properties** **of** **Linear/Affine** **Encoders).** *For each i=1,2, we have the following:*

*(a)*  *For any $x_{i}^{n},v_{i}^{n}\in X_{i}^{n}$ with $x_{i}^{n}\neq v_{i}^{n}$, we have*
  (17) $$\begin{array}{l} \mathrm{Pr}\left[\phi_{i}^{\left(n\right)}\left(x_{i}^{n}\right)=\phi_{i}^{\left(n\right)}\left(v_{i}^{n}\right)\right]=\mathrm{Pr}\left[\left(x_{i}^{n}⊖v_{i}^{n}\right)A=0^{m_{i}}\right]={\left|X\right|}^{-m_{i}}. \end{array}$$
*(b)*  *For any $s_{i}^{n}\in X_{i}^{n}$, and for any ${\tilde{s}}_{i}^{m_{i}}\in X^{m_{i}}$, we have*
  (18) $$\begin{array}{l} \mathrm{Pr}\left[\varphi_{i}^{\left(n\right)}\left(s_{i}^{n}\right)={\tilde{s}}_{i}^{m_{i}}\right]=\mathrm{Pr}\left[s^{n}A_{i}⊕b_{i}^{m_{i}}={\tilde{s}}_{i}^{m_{i}}\right]={\left|X_{i}\right|}^{-m_{i}}. \end{array}$$
*(c)*  *For any $s_{i}^{n},t_{i}^{n}\in X_{i}^{n}$ with $s_{i}^{n}\neq t_{i}^{n}$, and for any ${\tilde{s}}_{i}^{m_{i}}\in X_{i}^{m_{i}}$, we have*
  (19) $$\begin{array}{ll} \mathrm{Pr}[\varphi_{i}^{\left(n\right)}\left(s_{i}^{n}\right)=\varphi_{i}^{\left(n\right)}\left(t_{i}^{n}\right)={\tilde{s}}_{i}^{m_{i}}] & =\mathrm{Pr}\left[s_{i}^{n}A_{i}⊕b_{i}^{m_{i}}=t_{i}^{n}A_{i}⊕b_{i}^{m_{i}}={\tilde{s}}_{i}^{m_{i}}\right]={\left|X_{i}\right|}^{-2m_{i}}. \end{array}$$

Proof of this lemma is found in [26]. We omit the proof.

We next define the decoder function $\psi_{i}^{\left(n\right)}:X_{i}^{m_{i}}\to X_{i}^{n},i=1,2.$ To this end, we define the following quantities.

**Definition** **6.** *For $x_{i}^{n}\in X_{i}^{n}$, we denote the entropy calculated from the type $P_{x_{i}^{n}}$ by $H(x_{i}^{n})$. In other words, for a type $P_{{\bar{X}}_{i}}\in P_{n}\left(X_{i}\right)$ such that $P_{{\bar{X}}_{i}}=P_{x_{i}^{n}}$, we define $H\left(x_{i}^{n}\right)=H\left({\bar{X}}_{i}\right)$*.

*Minimum Entropy Decoder*: For each i=1,2, and for $\phi_{i}^{\left(n\right)}\left(x_{i}^{n}\right)={\tilde{x}}_{i}^{m_{i}}$, we define the decoder function $\psi_{i}^{\left(n\right)}:X_{i}^{m_{i}}\to X_{i}^{n}$ as follows:

$$\psi_{i}^{\left(n\right)}\left({\tilde{x}}_{i}^{m_{i}}\right):=\left\{\begin{array}{cc} {\hat{x}}_{i}^{n} & \mathrm{if}\;\phi_{i}^{\left(n\right)}\left({\hat{x}}_{i}^{n}\right)={\tilde{x}}_{i}^{m_{i}}\;\mathrm{and}\;H\left({\hat{x}}_{i}^{n}\right)<H\left({\overset{ˇ}{x}}_{i}^{n}\right)\\ & \mathrm{for}\;\mathrm{all}\;{\overset{ˇ}{x}}_{i}^{n}\mathrm{such}\;\mathrm{that}\;\;\phi_{i}^{\left(n\right)}\left({\overset{ˇ}{x}}_{i}^{n}\right)={\tilde{x}}_{i}^{m_{i}},\;\mathrm{and}\;\;{\overset{ˇ}{x}}_{i}^{n}\neq {\hat{x}}_{i}^{n},\;\\ \mathrm{arbitrary} & \mathrm{if}\;\mathrm{there}\;\mathrm{is}\;\mathrm{no}\;\mathrm{such}\;{\hat{x}}_{i}^{n}\in X_{i}^{n}. \end{array}\right.$$

*Error Probability Bound:* In the following arguments, we let expectations based on the random choice of the affine encoders $\varphi_{i}^{\left(n\right)}i=1,2$ be denoted by E[·]. For, i=1,2, define

$$\Pi_{{\bar{X}}_{i}}\left(R_{i}\right):=e^{-n{\left[R_{i}-H\left({\bar{X}}_{i}\right)\right]}^{+}}.$$

Then, we have the following lemma.

**Lemma** **6.** *For each i=1,2, for any n and for any $P_{{\bar{X}}_{i}}\in P_{n}\left(X_{i}\right)$*,

$$E\left[\Xi_{{\bar{X}}_{i}}\left(\phi_{i}^{\left(n\right)},\psi_{i}^{\left(n\right)}\right)\right]\leq e{\left(n+1\right)}^{|X_{i}|}\Pi_{\bar{X}}\left(R_{i}\right).$$

Proof of this lemma is found in [26]. We omit the proof.

*Estimation of Approximation Error:* Define

Θ(R1,R2,φA(n)|pZK1K2n):=∑(a,k1n,k2n)∈MA(n)×X1n×X2npMA(n)Kn(a,k1n,k2n)×log[1+(enR1−1)pK1n|MA(n)(k1n|a)+(enR2−1)pK2n|MA(n)(k2n|a)+(enR1−1)(enR2−1)pK1nK2n|MA(n)(k1n,k2n|a).

Then, we have the following lemma.

**Lemma** **7.** *For i=1,2 and for any $n,m_{i}$ satisfying $\left(m_{i}/n\right)\log\left|X_{i}\right|$$\leq R_{i}$, we have*

(20) $$\begin{array}{l} E\left[D\;\;\left.\left.\left.\left(p_{{\tilde{K}}^{m_{1}}{\tilde{K}}^{m_{2}}|M_{A}^{\left(n\right)}}\right|\right|p_{V_{1}^{m_{1}}V_{2}^{m_{2}}}\right|p_{M_{A}^{\left(n\right)}}\right)\right]\leq \Theta \left(R_{1},R_{2},\varphi_{A}^{\left(n\right)}|p_{ZK_{1}K_{2}}^{n}\right). \end{array}$$

Proof of this lemma is given in Appendix D. From the bound (20) in Lemma (7), we know that the quantity $\Theta (R_{1},$ $R_{2},\varphi_{A}^{\left(n\right)}\left|p_{ZK_{1}K_{2}}^{n}\right)$ serves as an upper bound of the ensemble average of the conditional divergence $D(p_{{\tilde{K}}_{1}^{m_{1}}{\tilde{K}}_{2}^{m_{2}}|M_{A}^{\left(n\right)}}$ $\left|\right|p_{V_{1}^{m_{1}}V_{2}^{m_{2}}}\left|p_{M_{A}^{\left(n\right)}}\right).$

From Lemmas 4 and 7, we have the following corollary.

**Corollary** **2.** $$\begin{array}{ll} E\left[\Delta_{n}\left(\varphi_{1}^{\left(n\right)},\varphi_{2}^{\left(n\right)},\varphi_{A}^{\left(n\right)}|p_{X_{1}X_{2}}^{n},p_{ZK_{1}K_{2}}^{n}\right)\right] & \leq \Theta (R_{1},R_{2},\varphi_{A}^{\left(n\right)}|p_{ZK_{1}K_{2}}^{n}). \end{array}$$

*Existence of Good Code*${\left\{\left(\varphi_{i}^{\left(n\right)},\psi_{i}^{\left(n\right)}\right)\right\}}_{i=1,2}$:

From Lemma 6 and Corollary 2, we have the following lemma stating an existence of universal code ${\left\{\left(\varphi_{i}^{\left(n\right)},\psi_{i}^{\left(n\right)}\right)\right\}}_{i=1,2}$.

**Lemma** **8.** *There exists at least one deterministic code ${\left\{\left(\varphi_{i}^{\left(n\right)},\psi_{i}^{\left(n\right)}\right)\right\}}_{i=1,2}$ satisfying $\left(m_{i}/n\right)\log\left|X_{i}\right|\leq R_{i},i=1,2$, such that, for i=1,2 and for any $p_{{\bar{X}}_{i}}$$\in P_{n}\left(X_{i}\right)$*,

$$\begin{array}{ll} \Xi_{{\bar{X}}_{i}}\left(\phi_{i}^{\left(n\right)},\psi_{i}^{\left(n\right)}\right)\leq e{\left(n+1\right)}^{|X_{i}|} & \times \left\{1+{\left(n+1\right)}^{|X_{1}|}+{\left(n+1\right)}^{|X_{2}|}\right\}\Pi_{{\bar{X}}_{i}}\left(R_{i}\right). \end{array}$$

*Furthermore, for any $\varphi_{A}^{\left(n\right)}\in F_{A}^{\left(n\right)}\left(R_{A}\right)$, we have*

$$\begin{array}{ll} \Delta_{n}\left(\varphi_{1}^{\left(n\right)},\varphi_{2}^{\left(n\right)},\varphi_{A}^{\left(n\right)}|p_{X_{1}X_{2}}^{n},p_{ZK_{1}K_{2}}^{n}\right) & \leq \left\{1+{\left(n+1\right)}^{|X_{1}|}+{\left(n+1\right)}^{|X_{2}|}\right\}\Theta \left(R_{1},R_{2},\varphi_{A}^{\left(n\right)}|p_{ZK_{1}K_{2}}^{n}\right). \end{array}$$

Basically, we can prove Lemma 8 in the same way as to prove a similar lemma shown in [4]. The detailed proof is given in Appendix E.

**Proposition** **1.** *For any $R_{A},R_{1},R_{2}>0$, and any $p_{ZK_{1}K_{2}}$, there exist two sequences of mappings ${\left\{\left(\varphi_{i}^{\left(n\right)},\psi_{i}^{\left(n\right)}\right)\right\}}_{n=1}^{\infty },i=1,2$ such that, for i=1,2 and for any $p_{X_{i}}\in P\left(X_{i}\right)$, we have*

(21) $$\begin{array}{l} \frac{1}{n}\log|X_{i}^{m_{i}}|=\frac{m_{i}}{n}\log\left|X_{i}\right|\leq R_{i},\\ p_{e}\left(\phi_{i}^{\left(n\right)},\psi_{i}^{\left(n\right)}|p_{X_{i}}^{n}\right)\leq e{\left(n+1\right)}^{2|X_{i}|}\times \left\{1+{\left(n+1\right)}^{|X_{1}|}+{\left(n+1\right)}^{|X_{2}|}\right\}e^{-nE(R_{i}|p_{X_{i}})} \end{array}$$

*and, for any eavesdropper A with $\varphi_{A}$ satisfying $\varphi_{A}^{\left(n\right)}\in F_{A}^{\left(n\right)}\left(R_{A}\right)$, we have*

(22) $$\begin{array}{ll} \Delta^{\left(n\right)}\left(\varphi_{1}^{\left(n\right)},\varphi_{2}^{\left(n\right)},\varphi_{A}^{\left(n\right)}|p_{X_{1}X_{2}}^{n},p_{ZK_{1}K_{2}}^{n}\right) & \leq \left\{1+{\left(n+1\right)}^{|X_{1}|}+{\left(n+1\right)}^{|X_{2}|}\right\}\times \Theta \left(R_{1},R_{2},\varphi_{A}^{\left(n\right)}|p_{ZK_{1}K_{2}}^{n}\right). \end{array}$$

**Proof.**  By Lemma 8, there exists $(\varphi_{i}^{\left(n\right)},$ $\psi_{i}^{\left(n\right)}),i=1,2,$ satisfying $\left(m_{i}/n\right)\log\left|X_{i}\right|\leq R_{i}$, such that for i=1,2 and for any $p_{{\bar{X}}_{i}}$ $\in P_{n}\left(X_{i}\right)$,

(23) $$\begin{array}{l} \Xi_{{\bar{X}}_{i}}\left(\phi_{i}^{\left(n\right)},\psi_{i}^{\left(n\right)}\right)\leq e{\left(n+1\right)}^{|X_{i}|}\times \left\{1+{\left(n+1\right)}^{|X_{1}|}+{\left(n+1\right)}^{|X_{2}|}\right\}\Pi_{\bar{X}}\left(R_{i}\right). \end{array}$$

Furthermore, for any $\varphi_{A}^{\left(n\right)}\in F_{A}^{\left(n\right)}\left(R_{A}\right)$,

(24) $$\begin{array}{ll} \Delta_{n}\left(\varphi_{1}^{\left(n\right)},\varphi_{2}^{\left(n\right)},\varphi_{A}^{\left(n\right)}|p_{X_{1}X_{2}}^{n},p_{ZK_{1}K_{2}}^{n}\right) & \leq \left\{1+{\left(n+1\right)}^{|X_{1}|}+{\left(n+1\right)}^{|X_{2}|}\right\}\times \Theta \left(R_{1},R_{2},\varphi_{A}^{\left(n\right)}|p_{ZK_{1}K_{2}}^{n}\right). \end{array}$$

The bound (22) in Proposition 1 has already been proved in (24). Hence, it suffices to prove the bound (21) in Proposition 1 to complete the proof. On an upper bound of $p_{e}\left(\phi_{i}^{\left(n\right)},\psi_{i}^{\left(n\right)}|p_{X_{i}}^{n}\right),i=1,2$, we have the following chain of inequalities:

$$\begin{array}{ll} p_{e}\left(\phi_{i}^{\left(n\right)},\psi_{i}^{\left(n\right)}|p_{X_{i}}^{n}\right) & \overset{\left(a\right)}{\leq }e{\left(n+1\right)}^{|X_{i}|}\times \left\{1+{\left(n+1\right)}^{|X_{1}|}+{\left(n+1\right)}^{|X_{2}|}\right\}\times \sum_{p_{{\bar{X}}_{i}}\in P_{n}\left(X_{i}\right)}\Pi_{{\bar{X}}_{i}}\left(R_{i}\right)e^{-nD(p_{{\bar{X}}_{i}}||p_{X_{i}})}\\ & \leq e{\left(n+1\right)}^{|X_{i}|}\left\{{\left(n+1\right)}^{|X_{i}|}+1\right\}\left|P_{n}\left(X_{i}\right)\right|e^{-nE(R_{i}|p_{X_{i}})}\\ & \overset{\left(b\right)}{\leq }e{\left(n+1\right)}^{2|X_{i}|}\left\{1+{\left(n+1\right)}^{|X_{1}|}+{\left(n+1\right)}^{|X_{2}|}\right\}\times e^{-nE(R_{i}|p_{X_{i}})}. \end{array}$$

Step (a) follows from Lemma 3 and (23). Step (b) follows from Lemma 1 part (a). ☐

### 5.4. Explicit Upper Bound of $\Theta (R_{1},R_{2},\varphi_{A}^{\left(n\right)}|p_{ZK_{1}K_{2}}^{n})$

In this subsection, we derive an explicit upper bound of $\Theta (R_{1},R_{2},\varphi_{A}^{\left(n\right)}|p_{ZK_{1}K_{2}}^{n}),$ which holds for any eavesdropper A with $\varphi_{A}$ satisfying $\varphi_{A}^{\left(n\right)}\in F_{A}^{\left(n\right)}\left(R_{A}\right)$. Define

$$\begin{array}{ll} {℘}_{0}:=p_{M_{A}^{\left(n\right)}Z^{n}K_{1}^{n}K_{2}^{n}} & \left\{\begin{array}{ccc} R_{1} & \geq \frac{1}{n}\log\frac{1}{p_{K_{1}^{n}|M_{A}^{\left(n\right)}}\left(K_{1}^{n}|M_{A}^{\left(n\right)}\right)}-\eta & \mathrm{or}\\ R_{2} & \geq \frac{1}{n}\log\frac{1}{p_{K_{2}^{n}|M_{A}^{\left(n\right)}}\left(K_{2}^{n}|M_{A}^{\left(n\right)}\right)}-\eta_{2} & \mathrm{or}\\ R_{1}+R_{2} & \geq \frac{1}{n}\log\frac{1}{p_{K_{1}^{n}K_{2}^{n}|M_{A}^{\left(n\right)}}\left(K_{1}^{n},K_{2}^{n}|M_{A}^{\left(n\right)}\right)}-\eta_{3} & \end{array}\right\}. \end{array}$$

For i=1,2, define

$$\begin{array}{ll} {℘}_{i}:=p_{M_{A}^{\left(n\right)}Z^{n}K_{i}^{n}}\{ & R_{i}\geq \left.\frac{1}{n}\log\frac{1}{p_{K_{i}^{n}|M_{A}^{\left(n\right)}}\left(K_{i}^{n}|M_{A}^{\left(n\right)}\right)}-\eta_{i}\right\}. \end{array}$$

Furthermore, define

$$\begin{array}{l} {℘}_{3}:=p_{M_{A}^{\left(n\right)}Z^{n}K_{1}^{n}K_{2}^{n}}\{R_{1}+R_{2}\geq \left.\frac{1}{n}\log\frac{1}{p_{K_{1}^{n}K_{2}^{n}|M_{A}^{\left(n\right)}}\left(K_{1}^{n},K_{2}^{n}|M_{A}^{\left(n\right)}\right)}-\eta_{3}\right\}. \end{array}$$

By definition, it is obvious that

(25) $$\begin{array}{ll} {℘}_{0} & \leq \sum_{i=1}^{3}{℘}_{i}. \end{array}$$

We have the following lemma.

**Lemma** **9.** *For any $\eta_{i}>0,i=1,2,3$ and for any eavesdropper A with $\varphi_{A}$ satisfying $\varphi_{A}^{\left(n\right)}\in F_{A}^{\left(n\right)}\left(R_{A}\right)$, we have the following:*

(26)Θ(R1,R2,φA(n)|pZK1K2n)≤n(R1+R2)℘0+∑i=13e−nηi(27)≤n(R1+R2)∑i=13℘i+∑i=13e−nηi.

*Specifically, if $n\geq {\left[R_{1}+R_{2}\right]}^{-1}$, we have*

(28) $$\begin{array}{l} {\left(n\left[R_{1}+R_{2}\right]\right)}^{-1}\Theta \left(R_{1},R_{2},\varphi_{A}^{\left(n\right)}|p_{ZK_{1}K_{2}}^{n}\right)\leq \sum_{i=1}^{3}\left({℘}_{i}+e^{-n\eta_{i}}\right). \end{array}$$

**Proof.**  By (25), it suffices to show (26) to prove Lemma 9. We set

$$\begin{array}{ll} A_{R_{1},R_{2}}\left(K_{1}^{n},K_{2}^{n}|M_{A}^{\left(n\right)}\right) & :=\;\left(e^{nR_{1}}-1\right)p_{K_{1}^{n}|M_{A}^{\left(n\right)}}\left(K_{1}^{n}|M_{A}^{\left(n\right)}\right)+\left(e^{nR_{2}}-1\right)p_{K_{2}^{n}|M_{A}^{\left(n\right)}}\left(K_{2}^{n}|M_{A}^{\left(n\right)}\right)\\ & \;+\left(e^{nR_{1}}-1\right)\left(e^{nR_{2}}-1\right)p_{K_{1}^{n}K_{2}^{n}|M_{A}^{\left(n\right)}}\left(K_{1}^{n},K_{2}^{n}|M_{A}^{\left(n\right)}\right). \end{array}$$

Then, we have

(29) $$\begin{array}{ll} \Theta (R_{1},R_{2},\varphi_{A}^{\left(n\right)}|p_{ZK_{1}K_{2}}^{n}) & =E\left[\log\left\{1+A_{R_{1},R_{2}}\left(K_{1}^{n},K_{2}^{n}|M_{A}^{\left(n\right)}\right)\right\}\right]. \end{array}$$

We further observe the following:

(30) $$\begin{array}{ll} \left\{\begin{array}{cc} R_{1} & <\frac{1}{n}\log\frac{1}{p_{K_{1}K_{2}^{n}|M_{A}^{\left(n\right)}}\left(K^{n}|M_{A}^{\left(n\right)}\right)}-\eta_{1}\;\\ R_{2} & <\frac{1}{n}\log\frac{1}{p_{K_{1}K_{2}^{n}|M_{A}^{\left(n\right)}}\left(K^{n}|M_{A}^{\left(n\right)}\right)}-\eta_{2}\;\\ R_{1}+R_{2} & <\frac{1}{n}\log\frac{1}{p_{K_{1}K_{2}^{n}|M_{A}^{\left(n\right)}}\left(K^{n}|M_{A}^{\left(n\right)}\right)}-\eta_{3} \end{array}\right. & \Rightarrow A_{R_{1},R_{2}}\left(K_{1}^{n},K_{2}^{n}|M_{A}^{\left(n\right)}\right)<\sum_{i=1}^{3}e^{-n\eta_{i}}\\ & \overset{\left(a\right)}{\Rightarrow }\log\left\{1+A_{R_{1},R_{2}}\left(K_{1}^{n},K_{2}^{n}|M_{A}^{\left(n\right)}\right)\right\}\leq \sum_{i=1}^{3}e^{-n\eta_{i}}. \end{array}$$

Step (a) follows from log(1+a)≤a. We also note that

(31) $$\begin{array}{ll} \log\{1 & +\left(e^{nR_{1}}-1\right)p_{K_{1}^{n}|M_{A}^{\left(n\right)}}\left(K_{1}^{n}|M_{A}^{\left(n\right)}\right)+\left(e^{nR_{2}}-1\right)p_{K_{2}^{n}|M_{A}^{\left(n\right)}}\left(K_{2}^{n}|M_{A}^{\left(n\right)}\right)\\ & \;+\left(e^{nR_{1}}-1\right)\left(e^{nR_{2}}-1\right)\times p_{K_{1}^{n}K_{2}^{n}|M_{A}^{\left(n\right)}}\left(K_{1}^{n},K_{2}^{n}|M_{A}^{\left(n\right)}\right)\}\leq \log\left[e^{nR_{1}}e^{nR_{2}}\right]=n\left(R_{1}+R_{2}\right). \end{array}$$

From (29)–(31), we have the bound (26). ☐

On upper bound of ${℘}_{i},i=1,2,3$, we have the following lemma:

**Lemma** **10.** *For any η>0 and for any eavesdropper A with $\varphi_{A}$ satisfying $\varphi_{A}^{\left(n\right)}\in F_{A}^{\left(n\right)}\left(R_{A}\right)$, we have that for each i=1,2, we have ${℘}_{i}\leq {\tilde{℘}}_{i}$, where*

(32) $$\begin{array}{l} {\tilde{℘}}_{i}:=p_{M_{A}^{\left(n\right)}Z^{n}K_{i}^{n}}\left\{\begin{array}{ccc} 0 & \geq \frac{1}{n}\log\frac{{\hat{q}}_{i,M_{A}^{\left(n\right)}Z^{n}K_{i}^{n}}\left(M_{A}^{\left(n\right)},Z^{n},K_{i}^{n}\right)}{p_{M_{A}^{\left(n\right)}Z^{n}K^{n}}\left(M_{A}^{\left(n\right)},Z^{n},K_{i}^{n}\right)}-\eta_{i}, & \;\;(a)\\ 0 & \geq \frac{1}{n}\log\frac{Q_{i,Z^{n}}\left(Z^{n}\right)}{p_{Z^{n}}\left(Z^{n}\right)}-\eta_{i}, & \;\;(b)\\ R_{A} & \geq \frac{1}{n}\log\frac{Q_{i,Z^{n}|M_{A}^{\left(n\right)}}\left(Z^{n}|M_{A}^{\left(n\right)}\right)}{p_{Z^{n}}\left(Z^{n}\right)}-\eta_{i}, & \;\;(c)\\ R_{i} & \geq \frac{1}{n}\log\frac{1}{Q_{i,K_{i}^{n}|M_{A}}^{\left(n\right)}\left(K_{i}^{n}|M_{A}^{\left(n\right)}\right)}-\eta_{i} \end{array}\right\}+3e^{-n\eta_{i}} \end{array}$$

*and that for i=3, we have ${℘}_{3}\leq {\tilde{℘}}_{3}$, where*

(33) $$\begin{array}{l} {\tilde{℘}}_{3}:=p_{M_{A}^{\left(n\right)}Z^{n}K_{1}^{n}K_{2}^{n}}\left\{\begin{array}{ccc} 0 & \geq \frac{1}{n}\log\frac{{\hat{q}}_{3,M_{A}^{\left(n\right)}Z^{n}K_{1}^{n}K_{2}^{n}}\left(M_{A}^{\left(n\right)},Z^{n},K_{1}^{n},K_{2}^{n}\right)}{p_{M_{A}^{\left(n\right)}Z^{n}K_{1}^{n}K_{2}^{n}}\left(M_{A}^{\left(n\right)},Z^{n},K_{1}^{n}K_{2}^{n}\right)}-\eta_{3}, & \;\;(a)\\ 0 & \geq \frac{1}{n}\log\frac{Q_{3,Z^{n}}\left(Z^{n}\right)}{p_{Z^{n}}\left(Z^{n}\right)}-\eta_{3}, & \;\;(b)\\ R_{A} & \geq \frac{1}{n}\log\frac{{\tilde{Q}}_{3,Z^{n}|M_{A}^{\left(n\right)}}\left(Z^{n}|M_{A}^{\left(n\right)}\right)}{p_{Z^{n}}\left(Z^{n}\right)}-\eta_{3}, & \;\;(c)\\ R_{1}+R_{2} & \geq \frac{1}{n}\log\frac{1}{p_{K_{1}^{n}K_{2}^{n}|M_{A}^{\left(n\right)}}\left(K_{1}^{n},K_{2}^{n}|M_{A}^{\left(n\right)}\right)}-\eta_{3} \end{array}\right\}+3e^{-n\eta_{3}}. \end{array}$$

*The probability distributions appearing in the three inequalities (a), (b), and (c) in the right members of (32) have a property that we can select them arbitrary. In (a), we can choose any probability distribution ${\hat{q}}_{i,M_{A}^{\left(n\right)}Z^{n}K_{i}^{n}}$ on $M_{A}^{\left(n\right)}$$\times Z^{n}$$\times X_{i}^{n}$. In (b), we can choose any distribution $Q_{i,Z^{n}}$ on $Z^{n}$. In (c), we can choose any stochastic matrix ${\tilde{Q}}_{i,Z^{n}|M_{A}^{\left(n\right)}}$: $M_{A}^{\left(n\right)}$$\to Z^{n}$. The probability distributions appearing in the three inequalities (a), (b), and (c) in the right members of (33) have a property that we can select them arbitrary. In (a), we can choose any probability distribution ${\hat{q}}_{3,M_{A}^{\left(n\right)}Z^{n}K_{1}^{n}K_{2}^{n}}$ on $M_{A}^{\left(n\right)}$$\times Z^{n}$$\times X_{1}^{n}$$\times X_{2}^{n}$. In (b), we can choose any distribution $Q_{3,Z^{n}}$ on $Z^{n}$. In (c), we can choose any stochastic matrix ${\tilde{Q}}_{3,Z^{n}|M_{A}^{\left(n\right)}}$: $M_{A}^{\left(n\right)}$$\to Z^{n}$*.

The above lemma is the same as Lemma 10 in the previous work [26]. Since the proof of the lemma is in [26], we omit the proof of Lemma 10 in the present paper. We have the following proposition.

**Proposition** **2.** *For any $\varphi_{A}^{\left(n\right)}\in F_{A}^{\left(n\right)}\left(R_{A}\right)$ and any $n\geq {\left[R_{1}+R_{2}\right]}^{-1}$, we have*

(34) $$\begin{array}{l} {\left(n\left[R_{1}+R_{2}\right]\right)}^{-1}\Theta \left(R_{1},R_{2},\varphi_{A}^{\left(n\right)}|p_{ZK_{1}K_{2}}^{n}\right)\leq 15e^{-nF_{\min}\left(R_{A},R_{1},R_{2}|p_{ZK_{1}K_{2}}\right)}. \end{array}$$

**Proof:**  By Lemmas 9 and 10, we have for any

(35) $$\begin{array}{ll} {\left(n\left[R_{1}+R_{2}\right]\right)}^{-1}\Theta \left(R_{1},R_{2},\varphi_{A}^{\left(n\right)}|p_{ZK_{1}K_{2}}^{n}\right) & \leq \sum_{i=1}^{3}\left({\tilde{℘}}_{i}+e^{-n\eta_{i}}\right). \end{array}$$

The quantity ${\tilde{℘}}_{i}+e^{-n\eta_{i}},i=1,2,3$. is the same as the upper bound on the correct probability of decoding for one helper source coding problem in Lemma 1 in Oohama [11] (extended version). In a manner similar to the derivation of the exponential upper bound of the correct probability of decoding for one helper source coding problem, we can prove that, for any $\varphi_{A}^{\left(n\right)}\in F_{A}^{\left(n\right)}\left(R_{A}\right),$ there exist $\eta_{i}^{*},i=1,2,3$ such that for i=1,2,3, we have

(36) $$\begin{array}{l} {\tilde{℘}}_{i}+e^{-n\eta_{i}^{*}}\leq 5e^{-nF(R_{A},R_{i}|p_{ZK_{i}})}. \end{array}$$

From (35) and (36), we have that for any $\varphi_{A}^{\left(n\right)}\in F_{A}^{\left(n\right)}\left(R_{A}\right)$ and any $n\geq {\left[R_{1}+R_{2}\right]}^{-1}$,

$$\begin{array}{l} {\left(n\left[R_{1}+R_{2}\right]\right)}^{-1}\Theta \left(R_{1},R_{2},\varphi_{A}^{\left(n\right)}|p_{ZK_{1}K_{2}}^{n}\right)\leq 5\sum_{i=1}^{3}e^{-nF(R_{A},R_{i}|p_{ZK_{i}})}\leq 15e^{-nF_{\min}\left(R_{A},R_{1},R_{2}|p_{ZK_{1}K_{2}}\right)}, \end{array}$$

completing the proof. □

## 6. Alternative Formulation

Here, we show an alternative way to formulate the main problem we consider in this paper. Originally, we consider a problem of having a reliable and secure broadcasting communication in the presence of a side-channel adversary in the case where the sender uses one-time-pad encryption. We can also formulate it in a slightly more general way as follows.

**Figure 6:**
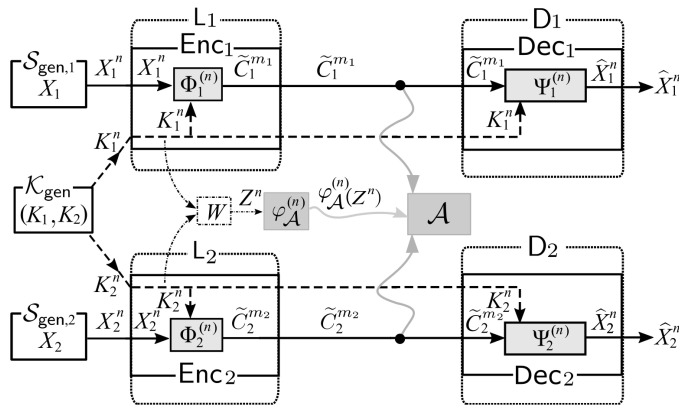
Broadcasting system AltSys from alternative formulation.

Let consider a problem of having a reliable and secure broadcasting communication in the presence of a side-channel adversary, in the case that the sender uses the encoding scheme $\Phi_{i}^{\left(n\right)}$ at node $L_{i}$, where $\Phi_{i}^{\left(n\right)}$ encodes $X_{i}^{\left(n\right)}$ and $K_{i}^{\left(n\right)}$ into ${\tilde{C}}_{i}^{\left(m_{i}\right)}$ for i=1,2. We denote the system resulted from the alternative formulation as AltSys. We illustrate AltSys in Figure 6.

### 6.1. Explanation on Sys and AltSys and Their Comparison

First, recall the “communication” channel *W* which is present in both systems, Sys and AltSys. The channel *W* represents the process of transforming analog raw physical data from the side-channel into raw digital data which later can be processed further by the side-channel adversary A.

In the broadcasting encryption system with post-encryption coding Sys shown in Figure 3, the *main* problem we consider to solve is how to strengthen the secrecy on broadcasting encrypted sources against side-channel adversary A, where the encryption function is one-time-pad encryption. In Sys, since the encryption has been explicitly described as one-time-pad encryption in the beginning, we always treat *W* as an immediate consequence of the side-channel attacks launched on one-time-pad encryption processes.

In the broadcasting system AltSys from our alternative formulation, shown in Figure 6, the problem we consider here is slightly different to the one in Sys. In AltSys, the problem we consider to solve is whether we can find or construct good encoding schemes that can guarantee the reliability and security against side-channel adversary A. In AltSys, we can have the properties of *W* fixed first, and then we will find good encoding schemes under the condition of the properties of *W*.

### 6.2. Reliability and Security of Alternative Formulation

We can also define the reliability and security of AltSys as follows in the same manner as the ones shown in Section 2.2.

*Defining Reliability and Security:* From the description of AltSys shown in Figure 6, the decoding process is successful if ${\hat{X}}_{i}^{n}=X_{i}^{n}$ holds. The decoding error probabilities $p_{e,i},i=1,2,$ are defined as follows:

$$\begin{array}{ll} p_{e,i}= & p_{e}\left(\Phi_{i}^{\left(n\right)},\Psi_{i}^{\left(n\right)}|p_{X_{i}}^{n},p_{K_{i}}^{n}\right):=\mathrm{Pr}\left[\Psi_{i}^{\left(n\right)}\left(\Phi_{i}^{\left(n\right)}\left(X_{i}^{n},K_{i}^{n}\right)\right)\neq X_{i}^{n}\right]. \end{array}$$

Recall that $X_{i}$ and $K_{i}$ are assumed to be independent. Let us set $M_{A}^{\left(n\right)}=\varphi_{A}^{\left(n\right)}\left(Z^{n}\right)$. The information leakage $\Delta^{\left(n\right)}$ on $\left(X_{1}^{n},X_{2}^{n}\right)$ from $\left({\tilde{C}}_{1}^{m_{1}},{\tilde{C}}_{2}^{m_{2}},M_{A}^{\left(n\right)}\right)$ is measured by the mutual information between $\left(X_{1}^{n},X_{2}^{n}\right)$ and $({\tilde{C}}_{1}^{m_{1}},{\tilde{C}}_{2}^{m_{2}},$ $M_{A}^{\left(n\right)})$. We can formally define this quantity by

$$\begin{array}{l} \Delta^{\left(n\right)}=\Delta^{\left(n\right)}\left(\Phi_{1}^{\left(n\right)},\Phi_{2}^{\left(n\right)},\varphi_{A}^{\left(n\right)}|p_{X_{1}X_{2}}^{n},p_{ZK_{1}K_{2}}^{n}\right):=I\left(X_{1}^{n}X_{2}^{n};{\tilde{C}}_{1}^{m_{2}},{\tilde{C}}_{2}^{m_{2}},M_{A}^{\left(n\right)}\right). \end{array}$$

**Definition** **7.** *A pair $\left(R_{1},R_{2}\right)$ is achievable under $R_{A}$>0 for the system AltSys if there exists two sequences $\{(\Phi_{i}^{\left(n\right)},$$\Psi_{i}^{\left(n\right)}{)\}}_{n\geq 1},i=1,2,$ such that ∀ϵ>0, $\exists n_{0}=n_{0}\left(\epsilon \right)\in N_{0}$, $\forall n\geq n_{0}$, we have for i=1,2,*

$$\begin{array}{l} \frac{1}{n}\log|X_{i}^{m_{i}}|=\frac{m_{i}}{n}\log\left|X_{i}\right|\leq R_{i},p_{e}\left(\Phi_{i}^{\left(n\right)},\Psi_{i}^{\left(n\right)}|p_{X_{i}}^{n},p_{K_{i}}^{n}\right)\leq \epsilon , \end{array}$$

*and for any eavesdropper A with $\varphi_{A}$ satisfying $\varphi_{A}^{\left(n\right)}\in F_{A}^{\left(n\right)}\left(R_{A}\right)$, we have*

$$\begin{array}{l} \Delta^{\left(n\right)}\left(\Phi_{1}^{\left(n\right)},\Phi_{2}^{\left(n\right)},\varphi_{A}^{\left(n\right)}|p_{X_{1}X_{2}}^{n},p_{ZK_{1}K_{2}}^{n}\right)\leq \epsilon . \end{array}$$

**Definition** **8** **(Reliable** **and** **Secure** **Rate** **Region).** *Let $R_{\mathrm{AltSys}}(p_{X_{1}X_{2}},$$p_{ZK_{1}K_{2}})$ denote the set of all $\left(R_{A},R\right)$ such that R is achievable under $R_{A}$. We call $R_{\mathrm{AltSys}}\left(p_{X_{1}X_{2}},p_{ZK_{1}K_{2}}\right)$ the***reliable and secure rate***region*.

**Definition** **9.** *A five tuple $\left(R_{1},R_{2},E_{1},E_{2},F\right)$ is achievable under $R_{A}>0$ for the system AltSys if there exists a sequence $\{(\Phi_{i}^{\left(n\right)},$$\Psi_{i}^{\left(n\right)}{)\}}_{n\geq 1}$, i=1,2, such that ∀ϵ>0, $\exists n_{0}=n_{0}\left(\epsilon \right)\in N_{0}$, ∀n$\geq n_{0}$, we have for i=1,2,*

$$\begin{array}{l} \frac{1}{n}\log|X_{i}^{m_{i}}|=\frac{m_{i}}{n}\log\left|X_{i}\right|\leq R_{i},p_{e}\left(\Phi_{i}^{\left(n\right)},\Psi_{i}^{\left(n\right)}|p_{X_{i}}^{n},p_{K_{i}}^{n}\right)\leq e^{-n(E_{i}-\epsilon )}, \end{array}$$

*and for any eavesdropper A with $\varphi_{A}$ satisfying $\varphi_{A}^{\left(n\right)}\in F_{A}^{\left(n\right)}\left(R_{A}\right)$, we have*

$$\begin{array}{l} \Delta^{\left(n\right)}\left(\Phi_{1}^{\left(n\right)},\Phi_{2}^{\left(n\right)},\varphi_{A}^{\left(n\right)}|p_{X_{1}X_{2}}^{n},p_{ZK_{1}K_{2}}^{n}\right)\leq e^{-n(F-\epsilon )}. \end{array}$$

**Definition** **10** **(Rate,** **Reliability,** **and** **Security** **Region).** *Let $D_{\mathrm{AltSys}}(p_{X_{1}X_{2}},$$p_{K_{1}K_{2}},W)$ denote the set of all $\left(R_{A},R,E,F\right)$ such that $\left(R_{1},R_{2},E_{1},E_{2},F\right)$ is achievable under $R_{A}$. We call $D_{\mathrm{AltSys}}(p_{X_{1}X_{2}},$$p_{K_{1}K_{2}},W)$ the***rate, reliability, and security***region*.

*Theoretical Results on the Reliable and Security for Broadcasting System from Alternative Formulation:* In order to provide solution for the problem from our alternative formulation, it is sufficient to show the existence of encoders and decoders $\{\left(\Phi_{i}^{\left(n\right)},\Psi_{i}^{\left(n\right)}\right)\},i=1,2$ which can guarantee reliable and security in the presence of a side-channel adversary. Based on the approach and theoretical results shown in Section 4 on proving the reliability and security of the broadcast system where the sender sends encrypted sources using one-time-pad encryption, it is easy to see that we can achieve the reliability and security for the broadcasting system from alternative formulation of the problem (Figure 6) such that the decoding error probabilities $p_{e,i}$ (i=1,2) and the information leakage $\Delta^{\left(n\right)}$ decay into zero in exponential rates by specifying $\Phi_{i}^{\left(n\right)}$ and $\Psi_{i}^{\left(n\right)}$, i=1,2, as follows:

(37) $$\begin{array}{lll} \Phi_{i}^{\left(n\right)}\left(X_{i}^{n},K_{i}^{n}\right) & :=\varphi_{i}^{\left(n\right)}\left({\mathrm{EncOTP}}_{i}^{\left(n\right)}\left(X_{i}^{n},K_{i}^{n}\right)\right) & \mathrm{for}\;i=1,2,\\ \Psi_{i}^{\left(n\right)}\left({\tilde{C}}_{i}^{m_{i}},K_{i}^{n}\right) & :=\psi_{i}^{\left(n\right)}\left({\mathrm{DecOTP}}_{i}^{\left(n\right)}\left({\tilde{C}}_{i}^{m_{i}},\varphi_{i}^{\left(n\right)}\left(K_{i}^{n}\right)\right)\right) & \mathrm{for}\;i=1,2, \end{array}$$

where:

- ${\mathrm{EncOTP}}_{i}^{\left(n\right)}:X_{i}^{n}\times X_{i}^{n}\to X_{i}^{n}$ is the one-time-pad encryption function defined as ${\mathrm{EncOTP}}_{i}^{\left(n\right)}\left(a,b\right):=a⊕b$ for $\left(a,b\right)\in X_{i}^{n}\times X_{i}^{n}$,
- $\varphi_{i}^{\left(n\right)}:X_{i}^{n}\to X_{i}^{m_{i}}$ is an affine encoder constructed based on a linear encoder $\phi_{i}^{\left(n\right)}:X_{i}^{n}\to X_{i}^{m_{i}}$ as shown in Section 5.3,
- ${\mathrm{DecOTP}}_{i}^{\left(n\right)}:X_{i}^{m_{i}}\times X_{i}^{m_{i}}\to X_{i}^{m_{i}}$ is the one-time-pad decryption function defined as ${\mathrm{DecOTP}}_{i}^{\left(n\right)}\left(a,b\right):=a⊖b$ for $\left(a,b\right)\in X_{i}^{m_{i}}\times X_{i}^{m_{i}}$,
- $\psi_{i}^{\left(n\right)}:X_{i}^{m_{i}}\to X_{i}^{n}$ is a decoder function for linear encoder $\phi_{i}^{\left(n\right)}$ which is associated with the affine encoder $\varphi_{i}^{\left(n\right)}$. (See Section 5.3 for the detailed construction.).

It is easy to see that Theorem 1 actually shows the achievability of reliability and security for broadcasting system in the presence of a side-channel adversary with the specification of $\Phi_{i}^{\left(n\right)}$ and $\Psi_{i}^{\left(n\right)}$, i=1,2 stated in Equation (37). Hence, the following theorem automatically holds.

**Theorem** **2.** *For any $R_{A},R_{1},R_{2}>0$ and any $p_{ZK_{1}K_{2}}$, there exist two sequences of mappings ${\left\{\left(\Phi_{i}^{\left(n\right)},\Psi_{i}^{\left(n\right)}\right)\right\}}_{n=1}^{\infty },i=1,2$ such that for any $p_{X_{i}}$ and $p_{K_{i}}$ for i=1,2, and any $n\geq {\left(R_{1}+R_{2}\right)}^{-1}$, we have*

(38) $$\begin{array}{ll} \frac{1}{n}\log\left|X_{i}^{m_{i}}\right| & =\frac{m_{i}}{n}\log\left|X_{i}\right|\leq R_{i},\\ p_{e}\left(\Phi_{i}^{\left(n\right)},\Psi_{i}^{\left(n\right)}|p_{X_{i}}^{n},p_{K_{i}}^{n}\right) & \leq e^{-n[E\left(R_{i}|p_{X_{i}}\right)-\delta_{i,n}]},i=1,2 \end{array}$$

*and for any eavesdropper A with $\varphi_{A}$ satisfying $\varphi_{A}^{\left(n\right)}\in F_{A}^{\left(n\right)}\left(R_{A}\right)$, we have*

(39) $$\begin{array}{ll} \Delta^{\left(n\right)}\left(\Phi_{1}^{\left(n\right)},\Phi_{2}^{\left(n\right)},\varphi_{A}^{\left(n\right)}|p_{X_{1}X_{2}}^{n},p_{K_{1}K_{2}}^{n},W^{n}\right) & \leq e^{-n[F_{\min}\left(R_{A},R_{1},R_{2}|p_{ZK_{1}K_{2}}\right)-\delta_{3,n}]}, \end{array}$$

*where $\delta_{i,n},i=1,2,3$ are defined by*

$$\begin{array}{ll} \delta_{i,n}:= & \frac{1}{n}\log\left[e{\left(n+1\right)}^{2|X_{i}|}\right.\times \left.\left\{1+{\left(n+1\right)}^{|X_{1}|}+{\left(n+1\right)}^{|X_{2}|}\right\}\right],for\;i=1,2,\\ \delta_{3,n}:= & \frac{1}{n}\log\left[15n\left(R_{1}+R_{2}\right)\times \left\{1+{\left(n+1\right)}^{|X_{1}|}+{\left(n+1\right)}^{|X_{2}|}\right\}\right]. \end{array}$$

*Note that, for i=1,2,3, $\delta_{i,n}\to 0$ as n→∞*.

It is easy to see that the proof of Theorem 1 that has been explained in Section 5 is also the proof of Theorem 2. Note that the functions $E(R_{i}|p_{X_{i}})$ and $F(R_{A},R_{1},R_{2}|p_{ZK_{1}K_{2}})$ take positive values if $\left(R_{A},R_{1},R_{2}\right)$ belongs to the set

$$\begin{array}{ll} R_{\mathrm{AltSys}}^{\left(\mathrm{in}\right)}\left(p_{X_{1}X_{2}},p_{ZK_{1}K_{2}}\right) & :=\left\{R_{1}>H\left(X_{1}\right)\right\}\cap \left\{R_{2}>H\left(X_{2}\right)\right\}\underset{i=1,2,3}{⋂}R_{i}^{c}\left(p_{ZK_{i}}\right). \end{array}$$

Then, define the following:

$$\begin{array}{ll} D_{\mathrm{AltSys}}^{\left(\mathrm{in}\right)}\left(p_{X_{1}X_{1}},p_{ZK_{1}K_{2}}\right) & :=\{\left(R_{A},R_{1},R_{2},E\left(R_{1}|p_{X_{1}}\right),E\left(R_{2}|p_{X_{2}}\right),F_{\min}\left(R_{A},R_{1},R_{2}|p_{K_{1}K_{2}}\right)\right):\\ & \;\left(R_{1},R_{2}\right)\in R_{\mathrm{Sys}}^{\left(\mathrm{in}\right)}\left(p_{X_{1}X_{2}},p_{ZK_{1}K_{2}}\right)\}. \end{array}$$

Hence, we have the following corollary.

**Corollary** **3.** $$\begin{array}{l} R_{\mathrm{AltSys}}^{\left(\mathrm{in}\right)}\left(p_{X_{1}X_{1}},p_{ZK_{1}K_{2}}\right)\subseteq R_{\mathrm{AltSys}}\left(p_{X_{1}X_{1}},p_{ZK_{1}K_{2}}\right),\\ D_{\mathrm{AltSys}}^{\left(\mathrm{in}\right)}\left(p_{X_{1}X_{1}},p_{ZK_{1}K_{2}}\right)\subseteq D_{\mathrm{AltSys}}\left(p_{X_{1}X_{1}},p_{ZK_{1}K_{2}}\right). \end{array}$$

## 7. Comparison to Previous Results

The following Table 1 shows the comparison between our result in this paper and already existing published research results which use the PEC paradigm for amplifying secrecy of the system.

## 8. Discussion on the Outer-Bounds of Rate Regions and Open Problems

In this paper, we have shown the inner-bound of $R_{\mathrm{Sys}}$ (resp. $R_{\mathrm{AltSys}}$). Although we have not touched the issue on the outer-bound of $R_{\mathrm{Sys}}$ (resp. $R_{\mathrm{AltSys}}$) in this paper, one may find the hints to derive the outer-bounds in Yamamoto [28]. However, it should be remarked that, in this paper, we are dealing with the side-channel adversary model, which is different from the wiretap model in Yamamoto [28]. In order to apply the method in Yamamoto [28] to find the outer-bound of $R_{\mathrm{Sys}}$ (resp. $R_{\mathrm{AltSys}}$), one may need to extend the method in Yamamoto [28] so that it can handle the rate constraint introduced by the side-channel adversary. We left the outer-bounds of $R_{\mathrm{Sys}}$ and $R_{\mathrm{AltSys}}$ as open problems.

Furthermore, in contrast to the case of $R_{\mathrm{Sys}}$ (resp. $R_{\mathrm{AltSys}}$) where we found hints in Yamamoto [28], we are not able to find any hints in the literature on determining the upper-bound of $D_{\mathrm{Sys}}$ (resp. $R_{\mathrm{AltSys}}$). We also left the outer-bounds of $D_{\mathrm{Sys}}$ (resp. $R_{\mathrm{AltSys}}$) as open problems.

## 9. Conclusions

In this paper, we have proposed a new model for analyzing the reliability and the security of broadcasting encrypted sources in the case of one-time-pad encryption, in the presence of an adversary that is not only eavesdropping the public communication channel to obtain ciphertexts but is also obtaining some physical information leaked by multiple devices owned by the sender while performing the encryption. We have also presented a countermeasure against such an adversary by utilizing affine encoders with certain properties. The main distinguishing feature of our countermeasure is that its performance is independent from the characteristics or the types of physical information leaked from the devices exploited by the adversary.

## Figures and Tables

**Table 1:** Comparison of research on application of PEC for secrecy amplification.

	Network System	Side-Channel Adversary	Correlated Keys
Previous work 1 [5,6]	Distributed Encryption (2 senders, 2 receivers)	No	Yes
Previous work 2 [4,7]	Two Terminals (1 sender, 1 receiver)	Yes	No
**This paper**	Broadcast Encryption (1 sender, 2 receivers)	**Yes**	**Yes**

## Appendix A. Computation of ${\;R}_{\mathrm{Sys},\mathrm{ex}1}^{\left(\mathrm{in}\right)}\left(p_{X_{1}X_{2}},p_{ZK_{1}K_{2}}\right)$

In this appendix, we compute the region $R_{\mathrm{Sys},\mathrm{ex}1}^{\left(\mathrm{in}\right)}\left(p_{X_{1}X_{2}},p_{ZK_{1}K_{2}}\right)$. Since $H\left(X_{i}\right)=h\left(s_{i}\right),i=1,2$, we have

(A1) $$\begin{array}{ll} R_{\mathrm{Sys}}^{\left(\mathrm{in}\right)}\left(p_{X_{1}X_{2}},p_{ZK_{1}K_{2}}\right) & =\left\{R_{1}>h\left(s_{1}\right)\right\}\cap \left\{R_{2}>h\left(s_{2}\right)\right\}\underset{i=1,2,3}{⋂}R_{i}^{c}\left(p_{ZK_{i}}\right). \end{array}$$

We compute $R(p_{ZK_{1}})$, $R(p_{ZK_{2}})$, and $R(p_{ZK_{1}K_{2}})$ explicitly. Then, we obtain the form of the region $R_{\mathrm{Sys},\mathrm{ex}1}^{\left(\mathrm{in}\right)}\left(p_{X_{1}X_{2}},p_{ZK_{1}K_{2}}\right)$ given by (13). We first compute $R(p_{ZK_{i}}),i=1,2$. Let ${\tilde{N}}_{A}$ be a binary random variable with $p_{{\tilde{N}}_{A}}\left(1\right)=\rho_{A}$. We assume that ${\tilde{N}}_{A}$ is independent from *Z*. Let *N* be a binary random variable with $p_{N}\left(1\right)=\rho$. We assume that *N* is independent from $\left(Z,{\tilde{N}}_{A}\right)$. Using ${\tilde{N}}_{A}$ and *N*, $K_{i},i=1,2$ can be written as

$$K_{1}=Z⊕{\tilde{N}}_{A},\;K_{2}=Z⊕{\tilde{N}}_{A}⊕N=K_{1}⊕N.$$

Then, by Example 10.2 (p. 265 in [24]), we have

(A2) $$\begin{array}{ll} R(p_{ZK_{1}}) & =\{\left(R_{A},R_{1}\right):R_{A}\geq \log2-h\left(\theta \right),R_{1}\geq h\left(\rho_{A}*\theta \right)\mathrm{for}\;\mathrm{some}\;\theta \in \left[0,1\right]\}, \end{array}$$

(A3) $$\begin{array}{ll} R(p_{ZK_{2}}) & =\{\left(R_{A},R_{2}\right):R_{A}\geq \log2-h\left(\theta \right),R_{2}\geq h\left(\rho *\rho_{A}*\theta \right)\mathrm{for}\;\mathrm{some}\;\theta \in \left[0,1\right]\}. \end{array}$$

We next compute $R(p_{ZK_{1}K_{2}})$. Note that

(A4) $$\begin{array}{l} H\left(K_{1}K_{2}|U\right)=H\left(K_{1}|U\right)+H\left(K_{2}|K_{1}U\right)\overset{\left(a\right)}{=}H\left(K_{1}|U\right)+H\left(K_{2}|K_{1}\right)\\ =H\left(K_{1}|U\right)+H\left(N\right)=H\left(K_{1}|U\right)+h\left(\rho \right). \end{array}$$

Step (a) follows from $U↔K_{1}↔K_{2}$. From (A4) and Example 10.2 (p. 265 in [24]), we have

(A5) $$\begin{array}{ll} R\left(p_{ZK_{1}K_{2}}\right)=\{\left(R_{A},R_{1},R_{2}\right):R_{A}\geq & \log2-h(\theta ),\\ R_{1}+R_{2}\geq & h\left(\rho \right)+h\left(\rho_{A}*\theta \right)\mathrm{for}\;\mathrm{some}\theta \in \left[0,1\right]\}. \end{array}$$

From (A1)–(A3) and (A5), we have the form of the region $R_{\mathrm{Sys},\mathrm{ex}1}^{\left(\mathrm{in}\right)}\left(p_{X_{1}X_{2}},p_{ZK_{1}K_{2}}\right)$ given by (13).

## Appendix B. Computation of ${\;R}_{\mathrm{Sys},\mathrm{ex}2}^{\left(\mathrm{in}\right)}\left(p_{X_{1}X_{2}},p_{ZK_{1}K_{2}}\right)$

In this appendix, we compute the region $R_{\mathrm{Sys},\mathrm{ex}2}^{\left(\mathrm{in}\right)}\left(p_{X_{1}X_{2}},p_{ZK_{1}K_{2}}\right)$. Since $H\left(X_{i}\right)=h\left(s_{i}\right),i=1,2$, we have

(A6) $$\begin{array}{ll} R_{\mathrm{Sys}}^{\left(\mathrm{in}\right)}\left(p_{X_{1}X_{2}},p_{ZK_{1}K_{2}}\right) & =\left\{R_{1}>h\left(s_{1}\right)\right\}\cap \left\{R_{2}>h\left(s_{2}\right)\right\}\underset{i=1,2,3}{⋂}R_{i}^{c}\left(p_{ZK_{i}}\right). \end{array}$$

We compute $R(p_{ZK_{1}})$, $R(p_{ZK_{2}})$, and $R(p_{ZK_{1}K_{2}})$ explicitly. Then, we obtain the form of the region $R_{\mathrm{Sys},\mathrm{ex}1}^{\left(\mathrm{in}\right)}\left(p_{X_{1}X_{2}},p_{ZK_{1}K_{2}}\right)$ given by (14). We first compute $R(p_{ZK_{i}}),i=1,2$. We can easily verify that for each i=1,2, $K_{i}$ is independent from *Z*. Then, for each i=1,2, we have

(A7) $$\begin{array}{ll} R(p_{ZK_{i}}) & =\{\left(R_{A},R_{i}\right):R_{A}\geq \log2-h\left(\theta \right),R_{i}\geq \log2\mathrm{for}\;\mathrm{some}\theta \in \left[0,1\right]\}. \end{array}$$

We next compute $R(p_{ZK_{1}K_{2}})$. To this end, we prove the following lemma.

**Lemma** **A1.**
*For Example 2, we have*

$$H\left(K_{1}K_{2}|U\right)=H\left(K_{2}\right)+H\left(K_{1}⊕K_{2}|U\right)=\log2+H\left(K_{1}⊕K_{2}|U\right).$$

**Proof.** Note that

(A8) $$\begin{array}{l} H\left(K_{1}K_{2}|U\right)=H\left(K_{1}+K_{2}|U\right)+H\left(K_{2}|K_{1}⊕K_{2},U\right)\\ =H\left(K_{1}⊕K_{2}|U\right)+H\left(K_{2}\right)-I\left(K_{2};K_{1}⊕K_{2},U\right). \end{array}$$

On the upper bound of $I(K_{2};K_{1}⊕K_{2},U)$, we have the following chain of inequalities:

(A9) $$\begin{array}{l} I\left(K_{2};K_{1}⊕K_{2},U\right)\leq I\left(K_{2};K_{1}⊕K_{2},U,Z\right)=I\left(K_{2};K_{1}⊕K_{2},Z\right)+I\left(K_{2};U|K_{1}⊕K_{2},Z\right)\\ \leq I\left(K_{2};K_{1}⊕K_{2},Z\right)+I\left(K_{1}⊕K_{2},K_{2};U|Z\right)\overset{\left(a\right)}{=}I\left(K_{2};N,N_{A}\right)+I\left(K_{1},K_{2};U|Z\right)\overset{\left(b\right)}{=}0. \end{array}$$

Step (a) follows from $K_{2}=K_{1}⊕N$ and $Z=K_{1}⊕K_{2}⊕N_{A}.$ Step (b) follows from $U↔Z↔(K_{1},K_{2}).$ From (A8) and (A9), we have Lemma A1. ☐

Let ${\hat{N}}_{A}$ be a binary random variable with $p_{{\hat{N}}_{A}}\left(1\right)=\rho_{A}$. We assume that ${\hat{N}}_{A}$ is independent from *Z*. Using ${\hat{N}}_{A}$, $X_{1}⊕X_{2}$ can be written as

(A10) $$\begin{array}{l} X_{1}⊕X_{2}=Z⊕{\hat{N}}_{A}. \end{array}$$

From Lemma A1, (A10), and Example 10.2 (p. 265 in [24]), we have

(A11) $$\begin{array}{ll} R\left(p_{ZK_{1}K_{2}}\right)=\{\left(R_{A},R_{1},R_{2}\right):R_{A} & \geq \log2-h(\theta ),\\ R_{1}+R_{2} & \geq \log2+h\left(\rho_{A}*\theta \right)\mathrm{for}\;\mathrm{some}\theta \in \left[0,1\right]\}. \end{array}$$

From (A6), (A7), and (A11), we have the form of the region $R_{\mathrm{Sys},\mathrm{ex}2}^{\left(\mathrm{in}\right)}\left(p_{X_{1}X_{2}},p_{ZK_{1}K_{2}}\right)$ given by (14).

## Appendix C. Proof of Lemma 4

We have the following chain of inequalities:

$$\begin{array}{ll} I({\tilde{C}}_{1}^{m_{1}}{\tilde{C}}_{2}^{m_{2}},M_{A}^{\left(n\right)};X_{1}^{n}X_{2}^{n}) & \overset{\left(a\right)}{=}I\left({\tilde{C}}_{1}^{m_{2}}{\tilde{C}}_{2}^{m_{2}};X_{1}^{n}X_{2}^{n}|M_{A}^{\left(n\right)}\right)\\ & \leq \log(|X_{1}^{m_{1}}\left|\right|X_{2}^{m_{2}}\left|\right)-H\left({\tilde{C}}_{1}^{m_{1}}{\tilde{C}}_{2}^{m_{2}}|X_{1}^{n}X_{2}^{n},M_{A}^{\left(n\right)}\right)\\ & \overset{\left(b\right)}{=}\log(|X_{1}^{m_{1}}\left|\right|X_{2}^{m_{2}}\left|\right)-H\left({\tilde{K}}_{1}^{m_{1}}{\tilde{K}}_{2}^{m_{2}}|X_{1}^{n}X_{2}^{n},M_{A}^{\left(n\right)}\right)\\ & \overset{\left(c\right)}{=}\log(|X_{1}^{m_{1}}\left|\right|X_{2}^{m_{2}}\left|\right)-H\left({\tilde{K}}_{1}^{m_{1}}{\tilde{K}}_{2}^{m_{2}}|M_{A}^{\left(n\right)}\right)\\ & =\left.\left.\left.\;\;D\left(p_{K_{1}^{m_{1}}K_{2}^{m_{2}}|M_{A}^{\left(n\right)}}\right|\right|p_{V_{1}^{m_{1}}V_{2}^{m_{2}}}\right|p_{M_{A}^{\left(n\right)}}\right). \end{array}$$

Step (a) follows from $\left(X_{1}^{n},X_{2}^{n}\right)⊥M_{A}^{\left(n\right)}$. Step (b) follows from that for i=1,2, ${\tilde{C}}_{i}^{m_{i}}={\tilde{K}}_{i}^{m_{i}}⊕{\tilde{X}}_{i}^{m_{i}}$ and ${\tilde{X}}_{i}^{m_{i}}=\phi_{i}^{\left(n\right)}\left(X_{i}^{n}\right)$. Step (c) follows from $({\tilde{K}}_{1}^{m_{1}},{\tilde{K}}_{2}^{m_{2}},$ $M_{A}^{\left(n\right)})⊥(X_{1}^{n},$ $X_{2}^{n})$.

## Appendix D. Proof of Lemma 7

In this appendix, we prove Lemma 7. This lemma immediately follows from the following lemma:

**Lemma** **A2.**
*For i=1,2 and for any $n,m_{i}$ satisfying $\left(m_{i}/n\right)\log\left|X_{i}\right|$$\leq R_{i},$ we have*

(A12) EDpK˜m1K˜m2|MA(n)pVm1Vm2pMA(n)≤∑(a,k1n,k2n)∈MA(n)×X1n×X2npMA(n)Kn(a,k1n,k2n)×log1+(|X1m1|−1)pK1n|MA(n)(k1n|a)+(|X2m2|−1)pK2n|MA(n)(k2n|a)+(|X1m1|−1)(|X2m2|−1)pK1nK2n|MA(n)(k1n,k2n|a).

In fact, from $|X_{i}^{m_{i}}|\leq e^{nR_{i}}$ and (A12) in Lemma A2, we have the bound (20) in Lemma 7. In this appendix we prove Lemma A2. In the following arguments, we use the following simplified notations:

$$\begin{array}{ll} k_{i}^{n},K_{i}^{n}\in X_{i}^{n} & ⟹k_{i},K_{i}\in K_{i}\\ {\tilde{k}}_{i}^{m_{i}},{\tilde{K}}_{i}^{m_{i}}\in X_{i}^{m_{i}} & ⟹l_{i},L_{i}\in L_{i}\\ \varphi_{i}^{\left(n\right)}:X_{i}^{n}\to X_{i}^{m_{i}} & ⟹\varphi_{i}:K_{i}\to L_{i}\\ \varphi_{i}^{\left(n\right)}\left(k_{i}^{n}\right)=k_{i}^{n}A_{i}+b_{i}^{m_{i}} & ⟹\varphi_{i}\left(k_{i}\right)=k_{i}A_{i}+b_{i}\\ V_{i}^{m_{i}}\in X_{i}^{m_{i}} & ⟹V_{i}\in L_{i}\\ M_{A}^{\left(n\right)}\in M_{A}^{\left(n\right)} & ⟹M\in M. \end{array}$$

We define

$$\chi_{l^{\prime },l}=\left\{\begin{array}{c} 1,\mathrm{if}l^{\prime }=l,\\ 0,\mathrm{if}l^{\prime }\neq l. \end{array}\right.$$

Then, the conditional distribution of the random pair $\left(L_{1},L_{2}\right)$ for given M=a∈M is

$$\begin{array}{l} p_{L_{1}L_{2}|M}\left(l|a\right)=\sum_{k\in K}p_{K_{1}K_{2}|M}\left(k_{1},k_{2}|a\right)\chi_{\varphi_{1}\left(k_{1}\right),l_{1}}\chi_{\varphi_{2}\left(k_{2}\right),l_{2}}\\ \mathrm{for}\left(l_{1},l_{2}\right)\in L_{1}\times L_{2}. \end{array}$$

Set

Υ(φ1(k1),l1),(φ2(k2),l2):=χφ1(k1),l1χφ2(k2),l2×log|L1||L2|∑(k1′,k2′)∈K1×K2pK1K2|M(k1′,k2′|a)χφ1(k1′),l1χφ2(k2′),l2.

Then, the conditional divergence between $p_{L_{1}L_{2}|M}$ and $p_{V_{1}V_{2}}$ for given *M* is given by

(A13) DpL1L2|MpV1V2pM=∑(a,k1,k2)∈M×K1×K2∑(l1,l2)∈L1×L2pMK1K2(a,k1,k2)Υ(φ1(k1),l1),(φ2(k2),l2).

The quantity $\Upsilon_{\left(\varphi_{1}\left(k_{1}\right),l_{1}\right),\left(\varphi_{2}\left(k_{2}\right),l_{2}\right)}$ has the following form:

(A14) Υ(φ1(k1),l1),(φ2(k2),l2)=χφ1(k1),l1χφ2(k2),l2×log|L1||L2|pK1K2|M(k1,k2|a)χφ1(k1),l1χφ2(k2),l2+∑k2′∈{k2}cpK1K2|M(k1,k2′|a)χφ1(k1),l1χφ2(k2′),l2+∑k1′∈{k1}cpK1K2|M(k1′,k2|a)χφ1(k1′),l1χφ2(k2),l2+∑(k1′,k2′)∈{k1}c×{k2}cpK1K2|M(k1′,k2′|a)χφ1(k1′),l1χφ2(k2′),l2.

The above form is useful for computing $E[\Upsilon_{\left(\varphi_{1}\left(k_{1}\right),l_{1}\right)}$ ${}_{,(\varphi_{2}\left(k_{2}\right),l_{2})}]$.

**Proof** **of** **Lemma** **A2.** Taking expectation of both side of (A14) with respect to the random choice of the entry of the matrix $A_{i}$ and the vector $b_{i}$ representing the affine encoder φ, we have

(A15) EDpL1L2|MpV1V2pM=∑(a,k1,k2)∈M×K1×K2∑(l1,l2)∈L1×L2pMK1K2(a,k1,k2)EΥ(φ1(k1),l1),(φ2(k2),l2).

To compute the expectation $E\left[\Upsilon_{\left(\varphi_{1}\left(k_{1}\right),l_{1}\right),\left(\varphi_{2}\left(k_{2}\right),l_{2}\right)}\right]$, we introduce an expectation operator useful for the computation. Let $E_{\varphi_{1}\left(k_{1}\right)=l_{k_{1}},\varphi_{2}\left(k_{2}\right)=l_{k_{2}}}\left[\cdot \right]$ be an expectation operator based on the conditional probability measures $\mathrm{Pr}\left(\cdot |\varphi_{1}\left(k_{1}\right)=l_{k_{1}},\varphi_{2}\left(k_{2}\right)=l_{k_{2}}\right)$. Using this expectation operator, the quantity $E\left[\Upsilon_{\left(\varphi_{1}\left(k_{1}\right),l_{1}\right),\left(\varphi_{2}\left(k_{2}\right),l_{2}\right)}\right]$ can be written as

(A16) EΥ(φ1(k1),l1),(φ2(k2),l2)=∑(lk1,lk2)∈L1×L2Prφ1(k1)=lk1,φ2(k2)=lk2×Eφ1(k1)=lk1,φ2(k2)=lk2Υ(lk1,l1),(lk2,l2).

Note that

(A17) $$\Upsilon_{\left(l_{k_{1}},l_{1}\right),\left(l_{k_{2}},l_{2}\right)}=\left\{\begin{array}{c} 1,\mathrm{if}\varphi_{1}\left(k_{1}\right)=l_{1},\varphi_{2}\left(k_{2}\right)=l_{2},\\ 0,\mathrm{otherwise}. \end{array}\right.$$

From (A16) and (A17), we have

(A18) $$\begin{array}{ll} E\left[\Upsilon_{\left(\varphi_{1}\left(k_{1}\right),l_{1}\right),\left(\varphi_{2}\left(k_{2}\right),l_{2}\right)}\right] & =\mathrm{Pr}\left(\varphi_{1}\left(k_{1}\right)=l_{1},\varphi_{2}\left(k_{2}\right)=l_{2}\right)\times E_{\varphi_{1}\left(k_{1}\right)=l_{1},\varphi_{2}\left(k_{2}\right)=l_{2}}\left[\Upsilon_{\left(l_{1},l_{1}\right),\left(l_{2},l_{2}\right)}\right]\\ & =\frac{1}{|L_{1}\left|\right|L_{2}|}E_{\varphi_{1}\left(k_{1}\right)=l_{1},\varphi_{2}\left(k_{2}\right)=l_{2}}\left[\Upsilon_{\left(l_{1},l_{1}\right),\left(l_{2},l_{2}\right)}\right]. \end{array}$$

Using (A14), the expectation $E_{\varphi_{1}\left(k_{1}\right)=l_{1},\varphi_{2}\left(k_{2}\right)=l_{2}}\left[\Upsilon_{\left(l_{1},l_{1}\right),(l_{2},}\right.$ $\left.{}_{l_{2})}\right]$ can be written as

(A19) Eφ1(k1)=l1,φ2(k2)=l2Υ(l1,l1),(l2,l2)=Eφ1(k1)=l1,φ2(k2)=l2log|L1||L2|pK1K2|M(k1,k2|a)+∑k2′∈{k2}cpK1K2|M(k1,k2′|a)χφ2(k2′),l2+∑k1′∈{k1}cpK1K2|M(k1′,k2|a)χφ1(k1′),l1+∑(k1′,k2′)∈{k1}c×{k2}cpK1K2|M(k1′,k2′|a)χφ1(k1′),l1χφ2(k2′),l2.

Applying Jensen’s inequality to the right member of (A19), we obtain the following upper bound of $E_{\varphi_{1}\left(k_{1}\right)=l_{1},\varphi_{2}(}$ ${}_{k_{2})=l_{2}}\left[\Upsilon_{\left(l_{1},l_{1}\right),\left(l_{2},l_{2}\right)}\right]$

(A20) Eφ1(k1)=l1,φ2(k2)=l2Υ(l1,l1),(l2,l2)≤log|L1||L2|pK1K2|M(k1,k2|a)+∑k2′∈{k2}cpK1K2|M(k1,k2′|a)E2+∑k1′∈{k1}cpK1K2|M(k1′,k2|a)E1+∑(k1′,k2′)∈{k1}c×{k2}cpK1K2|M(k1′,k2′|a)E12,

where we set

$$\begin{array}{ll} E_{1} & :=E_{\varphi_{1}\left(k_{1}\right)=l_{1},\varphi_{2}\left(k_{2}\right)=l_{2}}\left[\chi_{\varphi_{1}\left(k_{1}^{\prime }\right),l_{1}}\right],\\ E_{2} & :=E_{\varphi_{1}\left(k_{1}\right)=l_{1},\varphi_{2}\left(k_{2}\right)=l_{2}}\left[\chi_{\varphi_{2}\left(k_{2}^{\prime }\right),l_{2}}\right],\\ E_{12} & :=E_{\varphi_{1}\left(k_{1}\right)=l_{1},\varphi_{2}\left(k_{2}\right)=l_{2}}\left[\chi_{\varphi_{1}\left(k_{1}^{\prime }\right),l_{1}}\chi_{\varphi_{2}\left(k_{2}^{\prime }\right),l_{2}}\right]. \end{array}$$

Computing $E_{1}$, we have

(A21) $$\begin{array}{ll} E_{1} & =\mathrm{Pr}\left(\varphi_{1}\left(k_{1}^{\prime }\right)=l_{1}|\varphi_{1}\left(k_{1}\right)=l_{1},\varphi_{2}\left(k_{2}\right)=l_{2}\right)\overset{\left(a\right)}{=}\mathrm{Pr}\left(\varphi_{1}\left(k_{1}^{\prime }\right)=l_{1}|\varphi_{1}\left(k_{1}\right)=l_{1}\right)\overset{\left(b\right)}{=}\frac{1}{|L_{1}|}. \end{array}$$

Step (a) follows from that the random constructions of $\varphi_{1}$ and $\varphi_{2}$ are independent. Step (b) follows from Lemma 5 parts (b) and (c). In a similar manner we compute $E_{2}$ to obtain

(A22) $$\begin{array}{ll} E_{2} & =\frac{1}{|L_{2}|}. \end{array}$$

We further compute $E_{12}$ to obtain

(A23) $$\begin{array}{ll} E_{12} & =\mathrm{Pr}\left(\varphi_{1}\left(k_{1}^{\prime }\right)=l_{1},\varphi_{2}\left(k_{2}^{\prime }\right)=l_{2}\right.\left.|\varphi_{1}\left(k_{1}\right)=l_{1},\varphi_{2}\left(k_{2}\right)=l_{2}\right)\\ & \overset{\left(a\right)}{=}\mathrm{Pr}\left(\varphi_{1}\left(k_{1}^{\prime }\right)=l_{1}|\varphi_{1}\left(k_{1}\right)=l_{1}\right)\times \mathrm{Pr}\left(\varphi_{2}\left(k_{2}^{\prime }\right)=l_{2}|\varphi_{2}\left(k_{2}\right)=l_{2}\right)\overset{\left(b\right)}{=}\frac{1}{|L_{1}\left|\right|L_{2}|}. \end{array}$$

Step (a) follows from that the random constructuions of $\varphi_{1}$ and $\varphi_{2}$ are independent. Step (b) follows from Lemma 5 parts (b) and (c), From (A20)–(A23), we have

(A24) Eφ1(k1)=l1,φ2(k2)=l2Υ(l1,l1),(l2,l2)≤log|L1||L2|pK1K2|M(k1,k2|a)+∑k2′∈{k2}cpK1K2|M(k1,k2′|a)1|L2|+∑k1′∈{k1}cpK1K2|M(k1′,k2|a)1|L1|+∑(k1′,k2′)∈{k1}c×{k2}cpK1K2|M(k1′,k2′|a)1|L1||L2|=log1+(|L1|−1)pK1|M(k1|a)+(|L2|−1)pK2|M(k2|a)+(|L1|−1)(|L2|−1)pK1K2|M(k1,k2|a).

From (A15), (A18), and (A24), we have the bound (A12) in Lemma A2.

## Appendix E. Proof of Lemma 8

We have the following chain of inequalities:

$$\begin{array}{l} E\left[\frac{\Delta_{n}\left(\varphi_{1}^{\left(n\right)},\varphi_{2}^{\left(n\right)},\varphi_{A}^{\left(n\right)}|p_{X_{1}X_{2}}^{n},p_{ZK_{1}K_{2}}^{n}\right)}{\Theta (R_{1},R_{2},\varphi_{A}^{\left(n\right)}|p_{ZK_{1}K_{2}}^{n})}\right.+\sum_{i=1,2}\sum_{p_{{\bar{X}}_{i}}\in P_{n}\left(X_{i}\right)}\frac{\Xi_{{\bar{X}}_{i}}\left(\phi_{i}^{\left(n\right)},\psi_{i}^{\left(n\right)}\right)}{e{\left(n+1\right)}^{|X_{i}|}\Pi_{{\bar{X}}_{i}}\left(R_{i}\right)}\left.\begin{array}{c} \;\\ \;\\ \; \end{array}\;\;\right]\\ \;=\frac{E\left[\Delta_{n}\left(\varphi_{1}^{\left(n\right)},\varphi_{2}^{\left(n\right)},\varphi_{A}^{\left(n\right)}|p_{X_{1}X_{2}}^{n},p_{ZK_{1}K_{2}}^{n}\right)\right]}{\Theta (R_{1},R_{2},\varphi_{A}^{\left(n\right)}|p_{ZK_{1}K_{2}}^{n})}+\sum_{i=1,2}\sum_{p_{{\bar{X}}_{i}}\in P_{n}\left(X_{i}\right)}\frac{E\left[\Xi_{{\bar{X}}_{i}}\left(\phi_{i}^{\left(n\right)},\psi_{i}^{\left(n\right)}\right)\right]}{e{\left(n+1\right)}^{|X_{i}|}\Pi_{{\bar{X}}_{i}}\left(R_{i}\right)}\\ \;\overset{\left(a\right)}{\leq }1+\sum_{i=1,2}\sum_{p_{{\bar{X}}_{i}}\in P_{n}\left(X_{i}\right)}1\overset{\left(b\right)}{\leq }1+\sum_{i=1,2}{\left(n+1\right)}^{|X_{i}|}. \end{array}$$

Step (a) follows from Lemma 6 and Corollary 2. Step (b) follows from Lemma 1 part (a). Hence, there exists at least one deterministic code ${\left\{\left(\varphi_{i}^{\left(n\right)},\psi_{i}^{\left(n\right)}\right)\right\}}_{i=1,2}$ such that

$$\begin{array}{l} \frac{\Delta_{n}\left(\varphi_{1}^{\left(n\right)},\varphi_{2}^{\left(n\right)},\varphi_{A}^{\left(n\right)}|p_{X_{1}X_{2}}^{n},p_{ZK_{1}K_{2}}^{n}\right)}{\Theta (R_{1},R_{2},\varphi_{A}^{\left(n\right)}|p_{ZK_{1}K_{2}}^{n})}+\sum_{i=1,2}\sum_{p_{{\bar{X}}_{i}}\in P_{n}\left(X_{i}\right)}\frac{\Xi_{{\bar{X}}_{i}}\left(\phi_{i}^{\left(n\right)},\psi_{i}^{\left(n\right)}\right)}{e{\left(n+1\right)}^{|X_{i}|}\Pi_{{\bar{X}}_{i}}\left(R_{i}\right)}\leq 1+\sum_{i=1,2}{\left(n+1\right)}^{|X_{i}|}, \end{array}$$

from which we have that, for i=1,2 and for any $p_{{\bar{X}}_{i}}\in P_{n}\left(X_{i}\right)$,

$$\begin{array}{l} \frac{\Xi_{{\bar{X}}_{i}}\left(\phi_{i}^{\left(n\right)},\psi_{i}^{\left(n\right)}\right)}{e{\left(n+1\right)}^{|X_{i}|}\Pi_{{\bar{X}}_{i}}\left(R_{i}\right)}\leq 1+\sum_{j=1,2}{\left(n+1\right)}^{|X_{j}|}. \end{array}$$

Furthermore, we have that, for any $\varphi_{A}^{\left(n\right)}\in F_{A}^{\left(n\right)}\left(R_{A}\right)$,

$$\begin{array}{l} \frac{\Delta_{n}\left(\varphi_{1}^{\left(n\right)},\varphi_{2}^{\left(n\right)},\varphi_{A}^{\left(n\right)}|p_{X_{1}X_{2}}^{n},p_{ZK_{1}K_{2}}^{n}\right)}{\Theta (R_{1},R_{2},\varphi_{A}^{\left(n\right)}|p_{ZK_{1}K_{2}}^{n})}\leq 1+\sum_{j=1,2}{\left(n+1\right)}^{|X_{j}|}, \end{array}$$

completing the proof.
